# Caffeine Therapy for Apnea of Prematurity: Role of the Circadian *CLOCK* Gene Polymorphism

**DOI:** 10.3389/fphar.2021.724145

**Published:** 2022-01-25

**Authors:** Hong-Li Guo, Jia-Yi Long, Ya-Hui Hu, Yun Liu, Xin He, Ling Li, Ying Xia, Xuan-Sheng Ding, Feng Chen, Jing Xu, Rui Cheng

**Affiliations:** ^1^ Pharmaceutical Sciences Research Center, Department of Pharmacy, Children’s Hospital of Nanjing Medical University, Nanjing, China; ^2^ School of Basic Medical Sciences and Clinical Pharmacy, China Pharmaceutical University, Nanjing, China; ^3^ Neonatal Intensive Care Unit, Children’s Hospital of Nanjing Medical University, Nanjing, China

**Keywords:** preterm infant, apnea of prematurity, caffeine, circadian rhythm, clock, AHR, polymorphism

## Abstract

Standard-dose caffeine citrate has been routinely prescribed for apnea of prematurity (AOP) management; however, some preterm infants respond well to the therapy while others do not. The AOP phenotype has been attributed solely to the immature control of the respiratory system consequent to preterm birth, but there are also important genetic influences. Based on our previous report, we tested the hypothesis that the human circadian locomotor output cycles kaput (*CLOCK*) gene polymorphisms play a role in the response to caffeine citrate therapy in preterm infants. We also studied the interactions of the circadian clock with aryl hydrocarbon receptor (AHR) signaling pathways in preterm babies who received caffeine citrate. This single-center study collected data from 112 preterm infants (<35 weeks gestational age) between July 2017 and July 2018, including apnea-free (*n* = 48) and apneic (*n* = 64) groups. Eighty-eight candidate single nucleotide polymorphisms (SNPs) were tested using the MassARRAY system. Association analysis was performed using the PLINK Whole Genome Data Analysis Toolset and SNPStats software. Linkage disequilibrium (LD) and haplotype analyses were performed using Hapview software. No significant intergroup differences in allele distributions or genotype frequencies of *CYP1A2*, *CYP3A4*, *CYP3A5*, and *CYP3A7* were detected in our study on preterm babies. Two more SNPs in *AHR* were found to be associated with determining the response to caffeine citrate therapy in our pediatric patients. Of the 46 candidate SNPs in *the CLOCK* gene, 26 were found to be associated with determining the response to caffeine treatment in these babies. Interestingly, a significant association was retained for 18 SNPs in the *CLOCK* gene after false discovery rate correction. Moreover, strong LD formed in those variants in *AHR*, *ADORA2A*, and *CLOCK* genes was confirmed to be significantly associated with a better response to standard-dose caffeine therapy. In summary, *CLOCK* gene polymorphisms play a role in determining the response to caffeine therapy in premature neonates with AOP. However, whether the AHR and CLOCK signaling pathways crosstalk with each other during caffeine treatment remains largely unclear. Future clinical studies including more immature babies and basic research are needed to explore the mechanism by which circadian rhythms affect the response to caffeine therapy.

## Introduction

The WHO defines preterm birth as birth before 37 weeks of gestation. Premature babies, especially those born very early, often have complicated medical problems. A preterm baby may have trouble breathing due to an immaturely developed respiratory control system. For example, some preterm babies may experience a cessation of breathing for 20 s or longer or a shorter pause accompanied by bradycardia (<100 beats per minute), cyanosis, or pallor, known as apnea of prematurity (AOP) (Eichenwald et al., 2016). Neonates with AOP experience frequent episodes of apnea, resulting in hypoxemia and bradycardia, all of which place the infant at risk for prolonged mechanical ventilation, retinopathy of prematurity (ROP), bronchopulmonary dysplasia (BPD), and long-term neurodevelopmental impairment (Puia-Dumitrescu et al., 2019). Frequent apnea may be one of the most troublesome problems in the neonatal intensive care unit (NICU).

Various mechanisms implicated in the pathogenesis of AOP have been identified, at least in the central nervous system and peripheral reflex pathways (Eichenwald et al., 2016). Ventilatory responses to hypoxia and inhibitory reflexes were exaggerated in preterm neonates. These unique vulnerabilities predispose the neonate to the development of apnea (Mathew, 2011; Martin and Wilson, 2012). Importantly, AOP may also have a critical genetic basis underlying this development-related disorder of respiratory control (Bloch-Salisbury et al., 2010). Methylxanthines have been the mainstay of pharmacological therapy for AOP for over 40 years, and caffeine is generally preferred over other methylxanthines because of its wider therapeutic index and lower incidence of serious complications (Morton and Smith, 2016). However, caffeine is not completely efficient, and in about half of treated infants their apnea frequency remains elevated; thus, aggressive interventions such as mechanical ventilation are needed (Laouafa et al., 2019; He et al., 2021).

The blockade of inhibitory adenosine A_1_ receptors (A_1_-AR; encoded by the *ADORA1* gene), with the resultant respiratory neural output, as well as the blockade of excitatory adenosine A_2A_ receptors (A_2A_-AR; encoded by *ADORA2A* gene) located on γ-aminobutyric acidergic neurons has been recognized as the primary mechanism of action of caffeine therapy (Eichenwald et al., 2016). Caffeine also competitively inhibits phosphodiesterases (PDEs) and binds to intracellular calcium channel ryanodine receptors, leading to intracellular Ca^2+^ release (Kumar and Lipshultz, 2019). Specific *ADORA1* and *ADORA2A* polymorphisms have been associated with variability in response to caffeine therapy, as well as with a higher risk of AOP and BPD (Kumral et al., 2012). In our recent study, 88 single nucleotide polymorphisms (SNPs) in 19 genes encoding proteins involved in determining the disposition and pharmacological actions of caffeine were genotyped to evaluate the association between genetic mutations and the response to caffeine therapy in 112 preterm infants (He et al., 2021). The major findings of our previous study included the following: 1) No significant association between the plasma concentrations of caffeine and polymorphisms of caffeine-metabolism-related enzymes such as CYP1A2 and transcription factor aryl hydrocarbon receptor (AHR) was found; 2) polymorphisms of *ADORA1*, *ADORA2A*, *ADORA3*, and *PDE4D*, especially those of *AHR* and adenosine dehydrogenase (*ADA*) genes, play a critical role in determining interindividual variability to caffeine therapy. Interestingly, the influence of *AHR* polymorphisms on the response to caffeine therapy could not be explained by the mechanisms involving the AHR-CYP1A2 metabolic pathway (He et al., 2021). This AHR signaling pathway may play an important role in AOP development and the response to caffeine therapy.

In the respiratory control system, A_1_-ARs are found at high densities in the brainstem and anterior hypothalamus, while A_2A_-ARs are widely distributed in the medulla. Increased expression of *ADORA1* and *ADORA2A* was observed in both the brainstem and hypothalamus of caffeine-treated neonatal rats. Increased adenosinergic maturation in the central cardiorespiratory areas could partly explain the pharmacological effects of caffeine observed in premature infants (Gaytan and Pasaro, 2012). Adenosine is an inhibitory neuromodulator involved in sleep-wake regulation. A_2A_-ARs, but not A_1_-ARs, mediate the arousal effect of caffeine (Huang et al., 2005). Intriguingly, caffeine has been shown to influence circadian timing in humans by an A_1_-AR/cAMP-dependent mechanism (Burke et al., 2015). The basic mechanism of the clock is a cell-autonomous interlocked transcription-translation feedback loop sustained by transcriptional activators brain and muscle ARNT-Like 1 (BMAL1), also known as aryl hydrocarbon receptor nuclear translocator-like protein 1 (ARNTL1), Circadian Locomotor Output Cycles Kaput (CLOCK) or neuronal PAS domain protein (NPAS2), as well as period (PER) and cryptochrome (CRY) (Hardin and Panda, 2013). As the core molecular clock is present in nearly every cell in mammals (Mohawk et al., 2012), the circadian clock is likely involved in determining the tissue response to circulating factors, including drugs (Zhang et al., 2021). However, whether the human circadian clock affects the response to caffeine therapy in preterm infants is unknown.

Caffeine citrate was routinely prescribed for AOP management in the NICU. Some preterm infants respond well to caffeine, while others do not. We hypothesized that the human circadian clock is involved in the response to caffeine citrate therapy in preterm infants. The overarching goal of this study was to determine the association between circadian *CLOCK* gene polymorphisms in preterm babies and the clinical outcomes after caffeine treatment. In addition, this study touches on the crosstalk between the circadian clock and AHR signaling pathways based on our findings in preterm babies who received caffeine citrate treatment.

## Methods

A comprehensive description of the AOP, BPD, ROP, intraventricular hemorrhage (IVH), patent ductus arteriosus (PDA), and necrotizing enterocolitis (NEC), as well as the methods for determining the plasma caffeine concentration and collecting clinical data for a single-center, retrospective study in Chinese preterm infants has been published elsewhere (He et al., 2021). The caffeine citrate treatment protocol, clinical outcomes, and grouping criteria have also been clearly described (He et al., 2021). Briefly, the apneic group included patients with apnea episodes that occurred even once despite the combined use of necessary nonpharmacological therapies, and the apnea-free group included those without apnea following the administration of caffeine and necessary respiratory support options. The study protocol was approved by the hospital ethics committee of the Children’s Hospital of Nanjing Medical University (protocol number 201902082-1).

### Study Subjects

Fifty-five female and 57 male preterm babies were included in this study, of whom 104 were born at <32 weeks gestational age. Among these preterm babies, the median postmenstrual age was 29.3 weeks. The median birth weight was 1,265 *g*. The mean total duration of caffeine exposure was approximately 34 days. The median invasive intubation duration for respiratory support therapy in the 61 infants was 2 days, and the repeat intubation rate was approximately 26%. The use of nasal continuous positive airway pressure or supplemental oxygen therapy continued for a median of 26 days.

### MassArray SNP Detection

Because of the CLOCK pathways connected to the AHR pathway and the roles of caffeine in human circadian timing, we focused mainly on the following genes: *AHR* repressor (*AHRR*), aryl hydrocarbon receptor nuclear translocator (*ARNT*), *BMAL1*, and *CLOCK*. For *CLOCK* and *BMAL1* genes, SNPs associated with neuropsychiatric diseases and sleep homeostasis were selected as candidates. In addition, *CYP3A4*, *CYP3A5*, *CYP3A7*, and *CYP3A43*, especially *CYP3A7*, this isoform of CYP3A, has been demonstrated to metabolize endogenous compounds that are known to be important in the growth and development of fetuses and neonates. Ultimately, 88 relative SNPs were genotyped and evaluated in the current study.

The selected 88 SNPs in nine human genes were genotyped using the Agena MassARRAY platform 4.0 with iPLEX gold chemistry (Agena Bioscience, Inc., CA, United States). The main reagents contained Agena polymerase chain reaction (PCR) reagent, Agena shrimp alkaline phosphatase (SAP) reagent, and Agena iPLEX reagent. Genomic DNA was extracted from the peripheral blood cell sediment using a DNA extraction kit (BioTeke Corporation, Wuxi, China) according to the manufacturer’s instructions and stored at −80°C before genotyping. The purity and concentration of the extracted genomic DNA samples were assessed by absorbance measurements at 230, 260, and 280 nm using a NanoDrop 2000 UV-Vis spectrophotometer (Thermo Fisher Scientific, MA, United States). The Agena Bioscience Assay Designer 4.0 was used to design PCR amplification and extension primers for the 88 SNP variants. The primers used are listed in Supplementary Table S1. PCR master mixtures were obtained using the Agena PCR reagent set, the PCR procedures were started, and then the mixtures were treated with SAP. After extending the reaction, the samples underwent resin purification processing and dispensing on the 384-well SpectroCHIP bioarray using the MassARRAY Nanodispenser RS1000 spotter (Agena Bioscience, Inc., CA, United States), and the masses of the primer extension products correlated with genotype were then determined using matrix-assisted laser desorption/ionization time-of flight mass spectrometry (MALDI-TOF MS) (Agena Bioscience, Inc., CA, United States). The spectral profiles generated by MALDI-TOF MS were analyzed using MassARRAY TYPER 4.0 software (Agena Bioscience, Inc., CA, United States). Please refer to the study of Cheung and colleagues for a specific process (Cheung et al., 2016).

### Statistical Analysis

**FIGURE 1:**
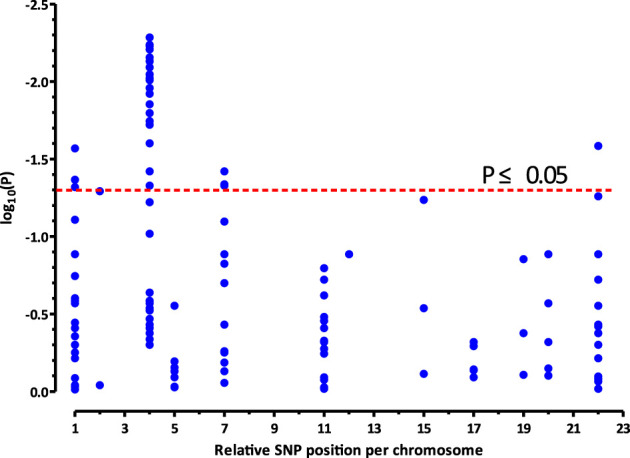
Significances (plotted as−log10 of nominal *p* values, closed blue circles) resulting from the associations between the investigated SNPs and different response (apnea-free *vs.* apneic) to caffeine therapy in preterm infants (*n* = 112). The *x*-axis represents the relative genomic position/chromosome, and the *y*-axis represents the distribution of the nominal−log10(*P*) values in the two groups of infants. The 26 SNPs, which are associated with the response to caffeine treatment (*p* < 0.05; above stippled red line), map to the *CLOCK* gene located on chromosome 4.

Statistical analyses were performed using SPSS version 25.0 software (IBM, Armonk, United States), and Figure 1 was drawn using GraphPad Prism 5 (GraphPad Software, CA, United States). The allele and genotype frequencies of various genes, including *CYP1A2, CYP3A4, CYP3A5, CYP3A7, AHR, AHRR, ARNT, BMAL1,* and *CLOCK*, were examined for deviation from the Hardy–Weinberg equilibrium (HWE) using the goodness-of-fit chi-square test. Distributions of genotype among neonates in the apneic and apnea-free groups were compared using the goodness-of-fit chi-square test. The Benjamini–Hochberg false discovery rate (FDR) was used for multiple hypothesis testing, and the adjusted *p* value (*p*_*FDR*_) was obtained. Multivariable logistic regression analysis was established through variable screening to explore the effect of significant outcomes from the univariate analysis of apneic and apnea-free groups. Statistical significance was set at *p* < 0.05.

The association between these tested SNPs and the response to caffeine citrate therapy was estimated by calculating odds ratios (ORs) and 95% confidence intervals (CIs) from logistic regression under the allele and recessive [AA *vs.* (CA + CC)], dominant [(CA + AA) *vs.* CC], additive (AA *vs* CC), over-dominant [(CC + AA) *vs* CA], and co-dominant (AA *vs* CA *vs* CC) models (A represents mutant allele and C represents wild type allele) using the PLINK Whole genome data analysis Toolset (version 1.07; http://zzz.bwh.harvard.edu/plink/tutorial.shtml) and SNPStats software (https://www.snpstats.net). Linkage disequilibrium (LD) and haplotype analyses were evaluated using the Hapview 4.2 software (The Broad Institute, MA, United States), while the difference in distribution of haplotype frequencies between the apneic and apnea-free groups was also tested using the goodness-of-fit chi-square analysis.

## Results

### Genotyping

In our previous study, we reported an association between 88 candidate SNPs in 19 human genes and the response to caffeine therapy (He et al., 2021). In this study, a total of 112 preterm babies were genotyped for 88 more SNPs in nine human genes*, CYP1A2*, *CYP3A4*, *CYP3A5*, *CYP3A7*, *AHR*, *AHRR*, *ARNT*, *BMAL1*, and *CLOCK*. The reference sequence (rs) numbers, alleles, call rate, minor allele frequencies (MAF), and HWE tests of the 176 SNPs included in this study are shown in Table 1. Eighty SNPs were common polymorphisms with MAFs of 1.4–50% and were in HWE (*p* > 0.01). Eight SNPs, including rs3805147 (*CLOCK*), rs3749474 (*CLOCK*), rs13132420 (*CLOCK*), rs1122780 (*BMAL1*), rs12504300 (*BMAL1*), rs2069514 (CYP1A2*1C), rs35694136 (CYP1A2*1D), and rs61469810 (CYP3A43*2A), were removed for further analysis because of low call rate and/or poor type clustering.

**TABLE 1:** Candidate SNPs for association with response to caffeine citrate therapy for AOP.

Gene	Location	SNP	Alleles	MAF	Call rate (%)	HWE	*p* ^e^
***ADORA1* (Adenosine A1 receptor)**	**1q32.1**	rs16851030	C/T	0.3519	100	0.054	0.39
		rs10920568	T/G	0.183	100	0.3581	0.13
		rs5780149	DEL/T	0.1045	97	1	0.44
		rs6427994	C/A	0.1514	97	1	0.26
		rs41264025	C/T	0.02232	100	1	0.27^a^
		rs6664108	T/C	0.2636	98	0.6221	0.93
		**rs12744240**	**G/T**	**0.1473**	**100**	**0.4586**	**0.043**
		rs3766553	A/G	0.192	100	0.02789	NA
		rs903361	G/A	0.3616	100	0.3146	0.82
		rs10920573	T/C	0.3773	98	0.07106	0.13
		rs6701725	A/G	0.1802	99	1	0.078
		rs3766566	G/A	0.3063	99	0.1799	0.36
		rs6677137	T/C	0.1636	98	0.03909	0.61
		rs3753472	T/C	0.08929	100	1	0.56
***ADORA2A* (Adenosine A2A receptor)**	**22q11.23**	rs5751862	G/A	0.455	99	1	0.13
		rs5760405^a^	C/T	<0.001	98	NONE	NONE
		rs2298383	C/T	0.473	100	1	0.42
		rs3761422	T/C	0.4364	100	0.4959	0.83
		rs2267076	T/C	0.4459	100	0.5104	0.8
		rs5751876	T/C	0.473	100	1	0.42
		rs4822492	C/G	0.4727	100	1	0.38
		rs5760423	T/G	0.473	100	1	0.42
		rs2236624	T/C	0.3348	100	0.671	0.19
		rs5760425	T/G	0.4775	98	0.8493	0.5
		rs4822498	T/C	0.486	95	0.175	0.38
		**rs34923252**	**T/A**	**0.08482**	**100**	**0.5654**	**0.026**
		**rs5996696**	**A/C**	**0.08482**	**100**	**0.5654**	**0.026**
		rs5760410	G/A	0.4633	97	0.8478	0.61
		rs4822489	T/G	0.4866	100	0.09115	0.86
		rs2236625	C/T	0.09009	99	1	0.055
*ADORA2B* (Adenosine A2B receptor)	17p12	rs2779193	A/G	0.02679	100	1	0.72^c^
		rs758857	G/A	0.2991	100	0.4986	0.51
		rs758858	A/G	0.02703	99	1	0.73^c^
		rs2041458	A/C	0.02679	100	1	0.72^c^
		rs17715109	G/T	0.06696	100	1	0.81^a^
		rs2015353	T/C	0.1545	98	1	0.48
		rs2779211	A/G	0.1545	98	1	0.48
***ADORA3* (Adenosine A3 receptor)**	**1p13.2**	**rs2298191**	**T/C**	**0.308**	**100**	**0.3736**	**0.048**
		**rs10776727**	**C/A**	**0.3818**	**98**	**0.5441**	**0.027**
		rs1544224^d^	A/G	NONE	100	NONE	NONE
		rs2229155	A/G	0.2545	98	0.449	0.5
		rs1544223	C/T	0.3468	99	0.6771	0.97
*ADA* (Adenosine deaminase)	20q13.12	rs73598374^d^	C/T	NONE	19	NONE	NONE
		rs6575353	G/A	0.4685	100	0.6544	0.48
		rs521704	C/A	0.4773	98	0.1794	0.79
		rs2472304	G/A	0.1577	100	1	0.13
		rs4998386^a^	C/T	<0.001	100	NONE	NONE
		rs382140	A/G	0.1111	97	0.6599	0.27
		rs9526558	A/G	0.1396	100	0.1998	0.71^a^
		rs17498920^a^	G/A	<0.001	100	NONE	NONE
***AHR* (Aryl hydrocarbon receptor)**	**7p21.1**	**rs4410790**	**T/C**	**0.4045**	**100**	**0.3618**	**0.047**
		**rs6968865**	**A/T**	**0.3739**	**100**	**0.8135**	**0.046**
		**rs2066853**	**G/A**	**0.3739**	**99**	**0.315**	**0.047**
		**rs1476080**	**T/G**	**0.3973**	**100**	**0.8459**	**0.038**
		rs10250822	C/T	0.4144	99	0.6992	0.55
		rs6960165	G/A	0.2027	99	0.1479	0.56
		rs7811989	A/G	0.2321	100	0.5991	0.2
		rs2158041	T/C	0.2321	100	0.2934	0.13
		rs10249788	C/T	0.2297	99	1	0.08
*AHRR* (Aryl-hydrocarbon receptor repressor)	5p15.33	rs2292596	C/G	0.3333		0.01834	0.93
*ARNT* (Aryl hydrocarbon receptor nuclear translocator)	1q21.3	rs2228099	C/G	0.4018	100	0.5547	0.91
*BMAL1* (Aryl hydrocarbon receptor nuclear translocator-like protein 1 or Brain and Muscle ARNT-Like 1)	11p15.3	rs11022775	C/T	0.125	96	1	0.84
		rs2290035	T/A	0.3191	84	0.2426	0.81
		rs3816358	C/A	0.2228	90	0.3877	0.39
		rs2278749	C/T	0.1963	96	0.06907	0.48
		rs3816360	T/C	0.398	88	0.1438	0.47
		rs1481892	G/C	0.4909	98	0.05564	0.35
		rs1122780^d^	A/G	NONE	73	NONE	NONE
		rs2279287	T/C	0.4954	97	0.03606	0.53
		rs4757144	G/A	0.4409	98	0.1766	0.57
		rs969485	G/A	0.4953	95	0.7002	0.96
		rs4757142	G/A	0.3423	99	0.676	0.94
		rs7126303	C/T	0.1759	96	0.313	0.33
		rs6486120	T/A	0.4771	97	1	0.16
		rs1868049	C/T	0.4554	90	0.1599	0.19
***CLOCK* (Circadian Locomotor Output Cycles Kaput)**	**4q12**	**rs1801260**	**A/G**	**0.08929**	**100**	**1**	**0.014**
		**rs2272073**	**C/T**	**0.4159**	**96**	**0.2317**	**0.018**
		rs6811520	T/C	0.3287	96	0.3815	0.5
		rs3762836	T/C	0.01429	94	1	0.06^a^
		**rs3805148**	**A/C**	**0.3952**	**94**	**0.2211**	**0.011**
		rs12504300^d^	G/C	NONE	94	NONE	NONE
		**rs12649507**	**G/A**	**0.4028**	**96**	**0.2291**	**0.0058**
		rs534654	A/G	0.01835	97	1	0.42^c^
		rs6850524	C/G	0.3178	96	0.8255	0.3
		**rs4340844**	**A/C**	**0.3991**	**97**	**0.1106**	**0.007**
		rs3736544	A/G	0.316	95	1	0.27
		**rs12648271**	**G/C**	**0.3962**	**95**	**0.3184**	**0.012**
		**rs2412648**	**T/G**	**0.3981**	**96**	**0.3141**	**0.0052**
		rs11735267	C/T	0.3165	97	1	0.34
		**rs7660668**	**C/G**	**0.08716**	**97**	**0.5761**	**0.0081**
		**rs10462028**	**G/A**	**0.07477**	**96**	**0.545**	**0.0081**
		**rs11932595**	**A/G**	**0.07944**	**96**	**0.4981**	**0.019**
		**rs2070062**	**A/C**	**0.09545**	**98**	**0.2452**	**0.016**
		rs13132420^d^	G/A	NONE	26	NONE	NONE
		rs11824092	T/C	0.4104	95	0.2289	0.39
		rs11931061	G/A	0.3108	99	1	0.26
		**rs11133385**	**G/A**	**0.3879**	**96**	**0.3074**	**0.0059**
		rs3749474^d^	C/T	NONE	87	NONE	NONE
		rs2412646	T/C	0.3066	95	1	0.39
		**rs11240**	**C/G**	**0.07477**	**96**	**0.454**	**0.0081**
		**rs3805151**	**T/C**	**0.3925**	**96**	**0.4172**	**0.0062**
		rs4580704	G/C	0.3102	96	1	0.3
		rs10002541	T/C	0.3148	96	1	0.3
		**rs4864546**	**G/A**	**0.3868**	**95**	**0.3077**	**0.0074**
		**rs6858749**	**T/C**	**0.1055**	**97**	**1**	**0.038**
		**rs6843722**	**A/C**	**0.4028**	**96**	**0.2291**	**0.0058**
		**rs4864548**	**G/A**	**0.3879**	**96**	**0.3074**	**0.0059**
		rs13102385	T/C	0.3165	97	1	0.34
		**rs7698022**	**G/T**	**0.08796**	**96**	**0.5798**	**0.009**
		**rs11133389**	**C/T**	**0.3952**	**94**	**0.4152**	**0.0098**
		rs11133373	C/G	0.4078	92	0.5397	0.096
		**rs11133391**	**T/C**	**0.3972**	**96**	**0.314**	**0.0095**
		**rs1048004**	**C/A**	**0.07477**	**96**	**0.454**	**0.0081**
		**rs11943456**	**T/C**	**0.09259**	**96**	**1**	**0.047**
		**rs3792603**	**A/G**	**0.075**	**89**	**0.4326**	**0.025**
		rs11726609	T/A	0.3131	96	1	0.23
		**rs17721497**	**A/T**	**0.07944**	**96**	**0.4981**	**0.019**
		rs3817444	A/C	0.3131	96	0.8234	0.26
		**rs9312661**	**G/A**	**0.3972**	**96**	**0.314**	**0.0095**
		rs726967	A/T	0.3194	96	0.661	0.3
		rs6832769	G/A	0.316	95	1	0.27
		rs3805147^d^	C/T	NONE	67	NONE	NONE
*CYP1A1*	15q24.1	rs2470893^a^	C/T	<0.001	100	NONE	NONE
*CYP1A2*		rs2472297^a^	C/T	<0.001	100	NONE	NONE
		rs2472299	A/G	0.3554	100	0.8039	0.29
		rs762551	C/A	0.3354	100	0.8039	0.29
CYP1A2*1B		rs2470890	T/C	0.1468	97	0.6976	0.058
CYP1A2*1C		rs2069514^d^	G/A	NONE	4	NONE	NONE
CYP1A2*1D		rs35694136^d^	T/DEL	NONE	0	NONE	NONE
CYP1A2*1E		rs2069526	T/G	0.1126	99	1	0.77
CYP2A6*7	19q13.2	rs5031016	A/G	0.1343	97	0.3429	0.78^a^
CYP2D6*2A	22q13.2	rs16947	G/A	0.15	100	0.06265	0.37^a^
CYP2D6*10		rs1065852	G/A	0.4909	100	1	0.96
		rs1135840	C/G	0.3257	98	0.04812	0.28
CYP3A4*1B	7q22.1	rs2740574**^b^**	C/T	<0.001	99	NONE	NONE
*CYP3A4* * *1G*		rs2242480	C/T	0.2252	100	1	0.88
CYP3A4*4		rs55951658^b^	T/C	<0.001	100	NONE	NONE
CYP3A4*18A		rs28371759^b^	A/G	<0.001	99	NONE	NONE
CYP3A4*23		rs2687116^b^	C/A	<0.001	95	NONE	NONE
CYP3A4*23		rs3735451	T/C	0.2432	99	0.6055	0.65
*CYP3A4*		rs4646437	G/A	0.08036	100	1	0.15
CYP3A5*4		rs56411402^b^	T/C	<0.001	100	NONE	NONE
CYP3A5*5		rs55965422^b^	A/G	<0.001		NONE	NONE
CYP3A7*1D		rs55798860^b^	C/T	<0.001	99	NONE	NONE
CYP3A7*2		rs2257401	G/C	0.2188	100	1	0.74
*CYP3A7*		rs10211^b^	T/A	<0.001	100	NONE	NONE
*CYP3A7*		rs12360	A/G	0.2315	96	1	0.65
CYP3A43*2A		rs61469810^d^	A/DEL	NONE	0	NONE	NONE
CYP3A43*3		rs680055^b^	C/G	<0.001	100	NONE	NONE
*PDE1A* (Phosphodiesterase)	2q32.1	rs1549870	G/A	0.2752	97	0.1531	0.91
*PDE1C*	7p14.3	rs30585	C/A	0.2232	100	1	0.37
		rs992185	C/A	0.2321	100	0.5991	0.2
*PDE2A*	11q13.4	rs341058	C/T	0.4107	100	0.5592	0.24
*PDE3A*	12p12.2	rs3794271	G/A	0.183	100	0.3581	0.13
		rs7134375	C/A	0.25	100	1	NA
*PDE4A*	19p13.2	rs6511698	C/T	0.491	99	0.572	0.14
		rs11670504^d^	A/G	NONE	58	NONE	NONE
		rs4804134	T/G	0.4775	99	0.8493	0.42
*PDE4B*	1p31.3	rs7537440^d^	T/G	NONE	98	NONE	NONE
		rs10454453	C/A	0.3705	100	0.6866	0.18
		rs783036	G/A	0.1205	100	0.3608	0.25
*PDE4D*	5q11.2-q12.1	rs966221	A/G	0.2432	99	0.6055	0.94
		rs702553	A/T	0.3784	99	0.5462	0.28
		rs16878037	C/T	0.1696	99	0.1898	0.64
		rs10075508	C/T	0.1696	100	0.5166	0.81
		rs829259	T/C	0.3125	100	0.1225	NA
		rs702531	A/C	0.2991	100	0.1123	0.74
		rs1823068^d^	A/G	NONE	25	NONE	NONE
		rs1588265	A/G	0.2909	98	1	0.7
*PDE5A*	4q26	rs12646525	T/C	0.1161	100	1	0.3
		rs3806808^d^	A/C	NONE	95	NONE	NONE
		rs11731756	T/G	0.344	97	0.6722	0.29
		rs10034450	A/G	0.4054	99	0.009987	0.46
		rs1155576	A/C	0.3468	99	0.8343	0.37
		rs3775843^d^	T/C	NONE	68	NONE	NONE
*PDE11A*	2q31.2	rs11684634	C/A	0.1027	100	0.3164	0.051

^a^No homozygous mutant, wild-type *vs.* heterozygous.

b No further statistical analysis for SNPs with MAF <0.001.

c No wild type, heterozygous *vs.* homozygous.

d Genotyping failure due to various reasons.

e Dominant model.

Abbreviations: NA, not available; MAF, minor allele frequencies.

Bold values represent statistically significant differences.

### Impact of Genetic Variability on the Clinical Response to caffeine Citrate Therapy

No significant intergroup differences in allele distributions or genotype frequencies of genes, *that is*, *CYP1A2*, *CYP3A4*, *CYP3A5*, and *CYP3A7*, encoding CYP450 proteins, were found in our study. Similar to our previous findings (He et al., 2021), two more SNPs (rs1476080 and rs2066853) (Table 2) in *AHR*, but not *AHRR* or *ARNT* genes, were found to be associated with the response to caffeine citrate therapy in our pediatric patients. Although the significance disappeared after FDR correction, the *AHR* polymorphism (rs1476080) still differed significantly (*p* = 0.033) between the two groups after adjusting for clinically important variables, including birth weight, birth height, respiratory support time, repeat intubation and caffeine therapy duration (Table 3).

**TABLE 2:** SNPs association with response to caffeine therapy among preterm infants in apneic and apnea-free groups^a^

Gene	SNPs	Genotype^b^	Frequency no. (%)	Apneic group no. (%)	Apnea-free group no. (%)	0dds ratio	95% CI	*p*_*chi-square*_	*p*_*FDR*_
*AHR*	*rs1476080 T > G*	TT	40 (35.71)	28 (43.8)	12 (25)	1		0.038	0.09938
		GT/GG	72 (64.29)	36 (56.2)	36 (75)	2.33	1.03–5.29		
	*rs2066853 G > A*	GG	46 (41.44)	21 (33.33)	25 (52.08)	1		0.047	0.1141
		GA/AA	65 (58.56)	42 (66.67)	23 (47.92)	0.46	0.21–0.99		
*CLOCK*	*rs1801260 A > G*	AA	93 (83.04)	58 (90.6)	35 (72.9)	1		0.014	0.05011
		AG/GG	19 (16.96)	6 (9.4)	13 (27.1)	3.59	1.25–1,031		
	***rs12649507 A > G***	**AA**	**35(32.41)**	**26 (43.3)**	**9 (18.8)**	**1**		**0.0058**	**0.04165**
		**GA/GG**	**73(67.59)**	**34 (56.7)**	**39 (81.2)**	**3.31**	**1.37–8.04**		
	*rs2070062 A > C*	AA	91 (82.73)	56 (90.3)	35 (72.9)	1		0.016	0.0544
		CA/CC	19 (17.27)	6 (9.7)	13 (27.1)	3.47	1.21–9.96		
	*rs2272073 T > C*	TT	33 (30.84)	24 (40)	9 (19.1)	1		0.018	0.05617
		CT/CC	74 (69.16)	36 (60)	38 (80.8)	2.81	1.15–6.86		
	***rs2412648 G > T***	**GG**	**36(33.33)**	**27 (44.3)**	**9 (19.1)**	**1**		**0.0052**	**0.04165**
		**GT/TT**	**72(66.67)**	**34 (55.7)**	**38 (80.8)**	**3.35**	**1.38–8.12**		
	***rs3805151 C > T***	**CC**	**37(34.58)**	**27 (45.8)**	**10 (20.8)**	**1**		**0.0062**	**0.04165**
		**CT/TT**	**70(65.42)**	**32 (54.2)**	**38 (79.2)**	**3.21**	**1.35–7.61**		
	***rs4340844 C > A***	**CC**	**35(32.11)**	**26 (42.6)**	**9 (18.8)**	**1**		**0.007**	**0.04165**
		**CA/AA**	**74(67.89)**	**35 (57.4)**	**39 (81.2)**	**3.22**	**1.33–7.80**		
	***rs6843722 C > A***	**CC**	**35(32.41)**	**26 (43.3)**	**9 (18.8)**	**1**		**0.0058**	**0.04165**
		**CA/AA**	**73(67.59)**	**34 (56.7)**	**39 (81.2)**	**3.31**	**1.37–8.04**		
	*rs6858749 C > T*	CC	87 (79.82)	53 (86.9)	34 (70.8)	1		0.038	0.9938
		CT/TT	22 (20.18)	8 (13.1)	14 (29.2)	2.73	1.03–7.19		
	***rs7660668 G > C***	**GG**	**91(83.49)**	**56 (91.8)**	**35 (72.9)**	**1**		**0.0081**	**0.04165**
		**CG/CC**	**18(16.51)**	**5 (8.2)**	**13 (27.1)**	**4.16**	**1.36–12.68**		
	***rs7698022 T > G***	**TT**	**90(83.33)**	**55 (91.7)**	**35 (72.9)**	**1**		**0.009**	**0.04165**
		**GT/GG**	**18(16.67)**	**5 (8.3)**	**13 (27.1)**	**4.09**	**1.34–12.46**		
	***rs10462028 G > A***	**GG**	**92(85.98)**	**58 (93.5)**	**34 (75.6)**	**1**		**0.0081**	**0.04165**
		**GA/AA**	**15(14.02)**	**4 (6.5)**	**11 (24.4)**	**4.69**	**1.38–15.89**		
	***rs1048004 C > A***	**CC**	**92(85.98)**	**58 (93.5)**	**34 (75.6)**	**1**		**0.0081**	**0.04165**
		**CA/AA**	**15(14.02)**	**4 (6.5)**	**11 (24.4)**	**4.69**	**1.38–15.89**		
	***rs11133385 A > G***	**AA**	**37(34.58)**	**28 (45.2)**	**9 (20)**	**1**		**0.0059**	**0.04165**
		**GA/GG**	**70(65.42)**	**34 (54.8)**	**36 (80)**	**3.29**	**1.36–7.98**		
	***rs11133389 T > C***	**TT**	**36(34.29)**	**27 (44.3)**	**9 (20.4)**	**1**		**0.0098**	**0.04165**
		**CT/CC**	**69(65.71)**	**34 (55.7)**	**35 (79.5)**	**3.09**	**1.27–7.52**		
	***rs11133391 C > T***	**CC**	**36(33.64)**	**27 (43.5)**	**9 (20)**	**1**		**0.0095**	**0.04165**
		**TC/TT**	**71(66.36)**	**35 (56.5)**	**36 (80)**	**3.09**	**1.27–7.49**		
	***rs11240 C > G***	**CC**	**92(85.98)**	**58 (93.5)**	**34 (75.6)**	**1**		**0.0081**	**0.04165**
		**CG/GG**	**15(14.02)**	**4 (6.5)**	**11 (24.4)**	**4.69**	**1.38–15.89**		
	*rs11932595 A > G*	AA	91 (85.05)	57 (91.9)	34 (75.6)	1		0.019	0.05617
		AG/GG	16 (14.95)	5 (8.1)	11 (24.4)	3.69	1.18–11.52		
	*rs11943456 C > T*	CC	89 (82.41)	55 (88.7)	34 (73.9)	1		0.047	0.1141
		TC/TT	19 (17.59)	7 (11.3)	12 (26.1)	2.77	0.99–7.73		
	***rs12648271 C > G***	**CC**	**36(33.96)**	**27 (43.5)**	**9 (20.4)**	**1**		**0.012**	**0.04533**
		**GC/GG**	**70(66.04)**	**35 (56.5)**	**35 (79.5)**	**3**	**1.23–7.29**		
	*rs17721497 A > T*	AA	91 (85.05)	57 (91.9)	34 (75.6)	1		0.019	0.05617
		TA/TT	16 (14.95)	5 (8.1)	11 (24.4)	3.69	1.18–11.52		
	*rs3792603 A > G*	AA	86 (86)	52 (92.9)	34 (77.3)	1		0.025	0.07083
		AG/GG	14 (14)	4 (7.1)	10 (22.7)	3.82	1.11–13.18		
	***rs3805148 C > A***	**CC**	**35(33.33)**	**26 (43.3)**	**9 (20)**	**1**		**0.011**	**0.044**
		**CA/AA**	**70(66.67)**	**34 (56.7)**	**36 (80)**	**3.06**	**1.25–7.46**		
	***rs4864546 A > G***	**AA**	**37(34.91)**	**28 (45.2)**	**9 (20.4)**	**1**		**0.0074**	**0.04165**
		**GA/GG**	**69(65.09)**	**34 (54.8)**	**35 (79.5)**	**3.2**	**1.32–7.78**		
	***rs4864548 A > G***	**AA**	**37(34.58)**	**28 (45.2)**	**9 (20)**	**1**		**0.0059**	**0.04165**
		**AG/GG**	**70(65.42)**	**34 (54.8)**	**36 (80)**	**3.29**	**1.36–7.98**		
	***rs9312661 A > G***	**AA**	**36(33.64)**	**27 (43.5)**	**9 (20)**	**1**		**0.0095**	**0.04165**
		**GA/GG**	**71(66.36)**	**35 (56.5)**	**36 (80)**	**3.09**	**1.27–7.49**		

a Data from apneic group was defined as case group and data from apnea-free group was defined as control group for the association analysis.

b Dominant model.

Abbreviations: *AHR*, aryl hydrocarbon receptor gene; CI, confidence interval; *CLOCK*, Circadian Locomotor Output Cycles Kaput.

**TABLE 3:** Results of multivariable logistic regression analysis to examine the effects of *AHR* and *CLOCK* gene polymorphisms on caffeine therapy in apnea-free group and apneic preterm infants.

SNPs		Constant	Birth weight - g	Birth height - cm	Respiration support time^a^ - day	Repeat intubation - no. (%)	Caffeine therapy duration - day	Gene mutation
rs1476080 (*AHR*)	B	2.964	0.003	−0.277	0.061	2.403	0.056	1.155
	*p*	0.338	0.027	0.018	0.012	0.039	0.009	**0.033**
	OR		1.003	0.758	1.063	11.054	1.058	3.173
	95% CI		1.00–1.01	0.60–0.95	1.01–1.12	1.13–107.88	1.01–1.10	1.10–9.18
rs1801260 (*CLOCK*)	B	1.874	0.003	−0.265	0.046	2.610	0.063	1.477
	*p*	0.545	0.034	0.022	0.045	0.039	0.005	**0.030**
	OR		1.003	0.767	1.047	13.596	1.065	4.381
	95% CI		1.00–1.01	0.61–0.96	1.00–1.10	1.14–162.53	1.02–1.11	1.15–16.69
rs12649507 (*CLOCK*)	B	1.789	0.002	−0.218	0.053	2.574	0.057	1.456
	*p*	0.576	0.123	0.068	0.026	0.031	0.011	**0.008**
	OR		1.002	0.804	1.055	13.121	1.058	4.289
	95% CI		1.00–1.01	0.64–1.02	1.01–1.11	1.26–136.63	1.01–1.11	1.45–12.68
rs2070062 (*CLOCK*)	B	1.717	0.003	−0.262	0.044	2.643	0.065	1.466
	*p*	0.580	0.032	0.024	0.057	0.037	0.004	**0.032**
	OR		1.003	0.769	1.045	14.049	1.067	4.331
	95% CI		1.00–1.01	0.61–0.97	1.00–1.09	1.18–167.57	1.02–1.12	1.13–16.54
rs2272073 (*CLOCK*)	B	1.807	0.002	−0.218	0.052	2.662	0.058	1.332
	*p*	0.571	0.113	0.066	0.028	0.023	0.010	**0.016**
	OR		1.002	0.804	1.054	14.326	1.059	3.788
	95% CI		1.00–1.01	0.64–1.01	1.01–1.10	1.44–142.89	1.01–1.11	1.28–11.18
rs2412648 (*CLOCK*)	B	3.960	0.002	−0.273	0.053	2.496	0.050	1.510
	*p*	0.243	0.106	0.028	0.027	0.037	0.023	**0.007**
	OR		1.002	0.761	1.055	12.139	1.052	4.527
	95% CI		1.00–1.01	0.60–0.97	1.01–1.11	1.17–126.32	1.01–1.10	1.51–13.60
rs3805151 (*CLOCK*)	B	2.091	0.002	−0.223	0.052	2.768	0.053	1.450
	*p*	0.515	0.129	0.062	0.029	0.018	0.018	**0.008**
	OR		1.002	0.800	1.053	15.919	1.054	4.261
	95% CI		1.00–1.01	0.63–1.01	1.01–1.10	1.60–158.05	1.01–1.10	1.46–12.41
rs4340844 (*CLOCK*)	B	1.738	0.002	−0.217	0.053	2.726	0.057	1.457
	*p*	0.588	0.126	0.071	0.026	0.020	0.011	**0.008**
	OR		1.002	0.805	1.055	15.273	1.058	4.293
	95% CI		1.00–1.01	0.64–1.02	1.01–1.11	1.53–152.67	1.01–1.11	1.45–12.71
rs6843722 (*CLOCK*	B	1.836	0.002	−0.216	0.052	2.724	0.055	1.461
	*p*	0.568	0.134	0.070	0.030	0.020	0.014	**0.008**
	OR		1.002	0.806	1.054	15.237	1.057	4.312
	95% CI		1.00–1.01	0.64–1.02	1.01–1.10	1.53–151.6	1.01–1.10	1.46–12.73
rs6858749 (*CLOCK*)	B	1.399	0.003	−0.233	0.041	2.580	0.066	1.326
	*p*	0.650	0.066	0.039	0.062	0.038	0.004	**0.043**
	OR		1.003	0.792	1.042	13.192	1.068	3.767
	95% CI		1.00–1.01	0.63–0.99	1.00–1.09	1.15–151.68	1.02–1.12	1.04–13.58
rs7660668 (*CLOCK*)	B	1.278	0.003	−0.246	0.039	2.772	0.068	1.826
	*p*	0.683	0.063	0.034	0.089	0.037	0.004	**0.016**
	OR		1.003	0.782	1.040	15.998	1.071	6.207
	95% CI		1.00–1.01	0.62–0.98	0.99–1.09	1.19–215.57	1.02–1.12	1.41–27.24
rs7698022 (*CLOCK*)	B	1.294	0.003	−0.245	0.039	2.653	0.068	1.809
	*p*	0.679	0.062	0.033	0.088	0.051	0.004	**0.016**
	OR		1.003	0.782	1.040	14.190	1.070	6.106
	95% CI		1.00–1.01	0.62–0.98	0.99–1.09	0.99–203.08	1.02–1.12	1.39–26.73
rs11133389 (*CLOCK*)	B	2.129	0.003	−0.230	0.054	2.559	0.051	1.387
	*p*	0.522	0.078	0.058	0.025	0.031	0.021	**0.010**
	OR		1.003	0.794	1.056	12.927	1.052	4.003
	95% CI		1.00–1.01	0.63–1.01	1.01–1.11	1.26–132.87	1.01–1.10	1.38–11.58
rs11932595 (*CLOCK*)	B	1.498	0.003	−0.265	0.043	3.203	0.064	2.090
	*p*	0.643	0.041	0.025	0.065	0.025	0.007	**0.013**
	OR		1.003	0.767	1.044	24.603	1.067	8.085
	95% CI		1.00–1.01	0.61–0.97	1.00–1.09	1.51–401.44	1.02–1.12	1.55–42.05
rs11943456 (*CLOCK*)	B	1.300	0.003	−0.243	0.044	2.577	0.065	1.564
	*p*	0.679	0.045	0.034	0.045	0.047	0.005	**0.028**
	OR		1.003	0.784	1.045	13.158	1.067	4.777
	95% CI		1.00–1.01	0.63–0.98	1.00–1.09	1.04–167.14	1.02–1.12	1.18–19.28
rs12648271 (*CLOCK*)	B	2.163	0.003	−0.233	0.055	2.561	0.051	1.388
	*p*	0.515	0.073	0.055	0.020	0.031	0.021	**0.011**
	OR		1.003	0.792	1.057	12.946	1.052	4.008
	95% CI		1.00–1.01	0.62–1.00	1.01–1.11	1.26–133.46	1.01–1.10	1.38–11.61
rs17721497 (*CLOCK*)	B	1.794	0.003	−0.267	0.045	2.631	0.061	1.731
	*p*	0.575	0.035	0.024	0.049	0.047	0.008	**0.021**
	OR		1.003	0.766	1.046	13.882	1.063	5.647
	95% CI		1.00–1.01	0.61–0.97	1.00–1.09	1.04–185.2	1.02–1.11	1.30–24.59
rs3792603 (*CLOCK*)	B	2.726	0.002	−0.265	0.033	3.034	0.071	2.241
	*p*	0.420	0.229	0.028	0.164	0.046	0.004	**0.023**
	OR		1.002	0.767	1.033	20.778	1.074	9.402
	95% CI		1.00–1.00	0.61–0.97	0.99–1.08	1.05–409.46	1.02–1.13	1.36–65.23
rs3805148 (*CLOCK*)	B	1.561	0.002	−0.204	0.053	2.606	0.053	1.442
	*p*	0.640	0.143	0.093	0.026	0.029	0.018	**0.009**
	OR		1.002	0.815	1.054	13.545	1.054	4.231
	95% CI		1.00–1.01	0.64–1.03	1.01–1.10	1.31–139.74	1.01–1.10	1.43–12.56
rs4864546 (*CLOCK*)	B	1.962	0.002	−0.222	0.055	2.602	0.051	1.518
	*p*	0.559	0.102	0.068	0.021	0.029	0.021	**0.006**
	OR		1.002	0.801	1.056	13.485	1.053	4.561
	95% CI		1.00–1.01	0.63–1.02	1.01–1.11	1.30–139.56	1.01–1.10	1.56–13.37
rs11133385	B	1.814	0.002	−0.219	0.056	2.616	0.052	1.543
	*p*	0.588	0.107	0.073	0.019	0.028	0.021	**0.005**
	OR		1.002	0.803	1.057	13.679	1.053	4.678
	95% CI		1.00–1.01	0.63–1.02	1.01–1.11	1.32–142.11	1.01–1.1	1.60–13.70
rs11133391	B	2.007	0.003	−0.230	0.057	2.575	0.051	1.413
	*p*	0.545	0.077	0.059	0.017	0.031	0.021	**0.009**
	OR		1.003	0.795	1.058	13.133	1.053	4.107
	95% CI		1.00–1.01	0.63–1.01	1.01–1.11	1.27–135.93	1.01–1.10	1.42–11.89
rs10462028	B	1.437	0.003	−0.267	0.042	2.840	0.066	2.239
rs1048004	*p*	0.656	0.040	0.023	0.067	0.047	0.006	**0.009**
rs11240 (*CLOCK*)	OR		1.003	0.765	1.043	17.124	1.068	9.386
	95% CI		1.00–1.01	0.61–0.96	1.00–1.09	1.04–280.82	1.02–1.12	1.77–49.9

a Duration for assisted ventilation through positive airway pressure or supplemental oxygen.

Abbreviations: *AHR*, aryl hydrocarbon receptor gene; *CLOCK*, Circadian Locomotor Output Cycles Kaput.

Most notably, in our 46 candidate SNPs in the *CLOCK* gene, excluding three unsuccessfully genotyped SNPs, 26 SNPs were found to be associated with the response to caffeine citrate treatment in these neonates (Table 2). Of these, 18 SNPs still differed significantly (*P*_*FDR*_<0.05) between the two groups after correction for multiple hypothesis testing. Furthermore, multivariable logistic regression analysis showed that the significant influence of these SNPs in the *CLOCK* gene on the response to caffeine therapy still survived after adjustment (Table 3). Intriguingly, no association was observed between the 14 candidate SNPs in the *BMAL1* gene, which encodes two essential components (*i.e.*, BMAL1 and CLOCK) of the circadian clock together with the *CLOCK* gene, and the clinical response to caffeine treatment.

In addition, some SNPs, especially for variants in *ADORA* genes, were found to be associated with the incidence rate of BPD and severe neurological injury (SNI) in these preterm infants. It is worth noting that significant associations between genetic polymorphisms of circadian rhythm-related genes, BPD, and SNI incidence, was also observed. The detailed results are shown in Supplementary Tables S2, S3.

### Haplotype and LD Analysis

**FIGURE 2:**
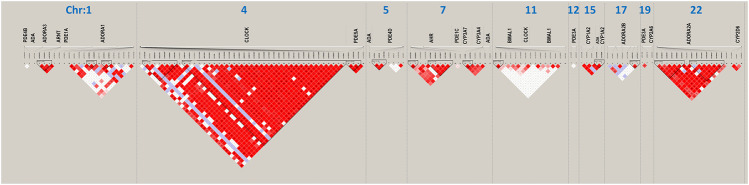
Linkage disequilibrium (LD) structure (triangle plots) for all investigated single nucleotide polymorphisms (SNPs) (rs numbers below gene names). Red diamonds indicate strong historical LD, *r* ^*2*^-based haplotype boundaries, among SNPs.

Haplotype analysis was performed to investigate whether various genetic variants were in LD. Fifteen SNPs were excluded because of failed genotyping, and 14 SNPs that were unable to meet the requirement for LD analysis were excluded, which are marked as ^d^ and ^b^ in Table 1, respectively. Along with our previous research (He et al., 2021), Figure 1 shows the distribution of the 147 candidate SNPs in more than 40 human genes evaluated in the present study. SNPs significantly associated (*p* < 0.05; above stippled line) with response to caffeine citrate therapy were *ADORA3*, *CLOCK*, *AHR*, and *ADORA2A*, which are located on chromosomes 1, 4, 7, and 22, respectively. We then compared *r* (Puia-Dumitrescu et al., 2019) (correlation coefficient between the two loci) and D’ (deviation of the observed frequency of a haplotype from the expected) values of the pairwise comparisons between the selected gene polymorphisms. The D’ and *r* (Puia-Dumitrescu et al., 2019) values were used for LD estimations. The results are shown in Figure 2. We observed two small blocks with strong LD between each pair of rs4410790 and rs6968865, and among variants rs1476080, rs6960165, rs2158041, rs7811989, and rs2066853 in the *AHR* gene, located on chromosome 7. Similarly, block 1 (rs5751862, rs5760410, rs2298383, rs3761422, rs5996696, and rs2267076), as well as block 2 (rs2236624, rs2236625, rs5751876, rs34923252, rs5760423, rs5760425, and rs4822492) with strong LD were found in the *ADORA2A* gene on chromosome 22. Most notably, 40 genetic *CLOCK* polymorphisms formed one small block (seven SNPs) and one big block (33 SNPs) on chromosome 4. More importantly, the results of haplotype analysis showed significant differences in the haplotypes, *that is*, TTAAAG (Block 1) in *CLOCK* (*p* = 0.0436), CT and TA (Block 1) (*p* = 0.00834), GACGG (Block 2) in *AHR* (*p* = 0.00243), GACCCC (Block 1) (*p* = 0.0401), and CTTATTC (Block 2) (*p* = 0.0284) in *ADORA2A*. Frequency distributions existed between these haplotypes and the response to caffeine citrate treatment (Table 4).

**TABLE 4:** Significant associated haplotype variants.

Block	NSNP^a^	NHAP^b^	SNP1^c^	SNP2^d^	HAPLOTYPE	F^e^	OR^f^	*P*
Chr4-Block1 (***CLOCK***)	**6**	**4**	**rs11943456**	**rs1801260**	**TTAAAG**	**0.0748**	**3.1**	**0.0436**
	6	4	rs11943456	rs1801260	TTGGCA	0.0187	0.376	0.409
	6	4	rs11943456	rs1801260	CCGGCA	0.297	1.09	0.784
	6	4	rs11943456	rs1801260	CCAGCA	0.604	0.685	0.232
Chr7-Block1 (***AHR***)	**2**	**3**	**rs4410790**	**rs6968865**	**CT**	**0.373**	**2.39**	**0.00834**
	2	3	rs4410790	rs6968865	CA	0.0318	0.944	0.943
	**2**	**3**	**rs4410790**	**rs6968865**	**TA**	**0.595**	**0.457**	**0.0122**
Chr7-Block2 (***AHR***)	5	4	rs1476080	rs2066853	TACGA	0.366	0.686	0.174
	5	4	rs1476080	rs2066853	TGTAG	0.196	0.763	0.43
	5	4	rs1476080	rs2066853	TATAG	0.0273	9.76E-60	0.994
	**5**	**4**	**rs1476080**	**rs2066853**	**GACGG**	**0.388**	**2.53**	**0.00243**
Chr22-Block1 (***ADORA***_***2A***_)	6	6	rs5751862	rs2267076	GGCTAT	0.413	1.05	0.856
	6	6	rs5751862	rs2267076	GACTAT	0.0142	8.65E-08	0.664
	**6**	**6**	**rs5751862**	**rs2267076**	**GACCCC**	**0.0811**	**2.95**	**0.0401**
	6	6	rs5751862	rs2267076	AGTCAC	0.0139	0.54	0.633
	6	6	rs5751862	rs2267076	GGTCAC	0.0273	1.25	0.797
	6	6	rs5751862	rs2267076	AATCAC	0.427	0.759	0.313
Chr22-Block2 (***ADORA***_***2A***_)	7	4	rs2236624	rs4822492	CCTTTTC	0.107	1.7	0.233
	7	4	rs2236624	rs4822492	TCTTTTC	0.326	0.675	0.205
	7	4	rs2236624	rs4822492	CCCTGGG	0.473	0.781	0.376
	**7**	**4**	**rs2236624**	**rs4822492**	**CTTATTC**	**0.0848**	**3.15**	**0.0284**

a Number of SNPs.

b Number of haplotype.

c Starting SNP

d Ending SNP

e Frequency of haplotype

f Odds ratio

## Discussion

The clear benefits and safety of caffeine citrate based on a standard-dose regimen have led to its wide and early use in very low birth weight infants. However, caffeine is not completely efficient, and in approximately 50% of treated infants, the apnea frequency remains elevated (Laouafa et al., 2019; He et al., 2021). In fact, studies are warranted to better understand why some preterm neonates persist with apnea after caffeine therapy, even after increasing the caffeine dose. The AOP phenotype has been attributed solely to immature respiratory system control consequent to preterm birth, but there may also be important genetic influences (Erickson et al., 2021). Therefore, the influence of genetics on the efficacy of caffeine in preterm infants should be better explored (Long et al., 2021).

In the present study, genetic polymorphisms of *CYP3A* genes, including *3A4*, *3A5*, *3A7*, and *3A43*, were also assessed (Table 1). Especially for CYP3A7, this isoform of CYP3A has been demonstrated to metabolize endogenous compounds that are known to be important in the growth and development of the fetus and neonate. In addition, CYP3A7 is an important component in the development and protection of the fetal liver and plays a role in certain disease status (Li and Lampe, 2019). However, because of MAF values < 0.001 or genotyping failure (Table 1), most of the selected SNPs were excluded from further association analysis.

A major finding of this study was that *AHR* genetic variations (rs1476080 and rs2066853), but not *AHRR* or *ARNT* genes, were found to be associated with the response to caffeine therapy (Tables 2, 3). Our previous study showed that two particular polymorphisms of the *AHR* gene (rs6968865 and rs4410790) were significantly associated with the response to caffeine treatment between the two groups of preterm infants (He et al., 2021). Furthermore, these variants in the *AHR* gene, located on chromosome 7, formed strong LD, with increased ORs for haplotypes CT (*p* = 0.00834), TA (*p* = 0.0122), and GACGG (*p* = 0.00243), which in part determined a better response to caffeine treatment (Figure 2; Table 4). Based on the plasma concentration data of caffeine, we excluded that the AHR-CYP1A2 metabolic pathway was responsible for the variable response to caffeine therapy (He et al., 2021). However, AHR is necessary to protect fetal human pulmonary microvascular endothelial cells against hyperoxic injury (Zhang et al., 2015) and plays a critical role in the maintenance of lung health (Guerrina et al., 2018). Therefore, the findings of our study suggest the potential role of AHR signaling in preterm infants who experience AOP episodes. The AHR signaling pathway may act alone or in combination with adenosine receptors and circadian CLOCK.

Another important finding in the present study was the significant association between circadian *CLOCK* polymorphisms, but not *BMAL1* variants (also known as *ARNTL1*; Table 1), and the response to caffeine citrate therapy in these preterm babies (Table 2). Notably, after adjustment for clinically important variables, such as birth weight, birth height, respiratory support therapy, repeat intubation, and caffeine therapy duration, the significance was retained (Table 3). Moreover, 40 candidate *CLOCK* SNPs formed one small block and one big block with strong LD (Figure 2). Especially for the small block, the haplotype TTAAAG was found to be associated with a better response to caffeine therapy (OR = 3.1, *p* = 0.0436; Table 4).

Caffeine has been reported to affect the phase of the human circadian clock and primarily affect human cellular circadian clocks via an A_1_-R/cAMP-dependent mechanism (Burke et al., 2015). Recent studies have revealed that caffeine and adenosine alter clock gene expression and circadian rhythms *in vitro* and *in vivo* via the Ca^2+^–ERK–AP-1 pathway (Jagannath et al., 2021). The present study is the first to show that genetic polymorphisms of *CLOCK*, encoding one of the two core components of the circadian rhythm, were significantly associated with the response to caffeine therapy. These findings suggest that the circadian rhythm may play critical roles in the response to caffeine citrate therapy in babies experiencing AOP episodes. Interestingly, caffeine has been reported to increase the light responsiveness of the mouse circadian pacemaker (van Diepen et al., 2014) and also affects the human circadian clock *in vivo* and *in vitro* (Burke et al., 2015). Therefore, synchronizing caffeine with the circadian rhythm may be useful for optimizing its treatment efficacy. In the meantime, investigations into essential mechanisms may provide therapies to reset or amplify circadian signals. However, very few studies have been performed to evaluate the proper timing of methylxanthine dosing (chronotherapy) as a means to maximize its efficacy and possibly reduce its adverse effects. Further research in this field is warranted to provide new insights and clinical advantages.

**FIGURE 3:**
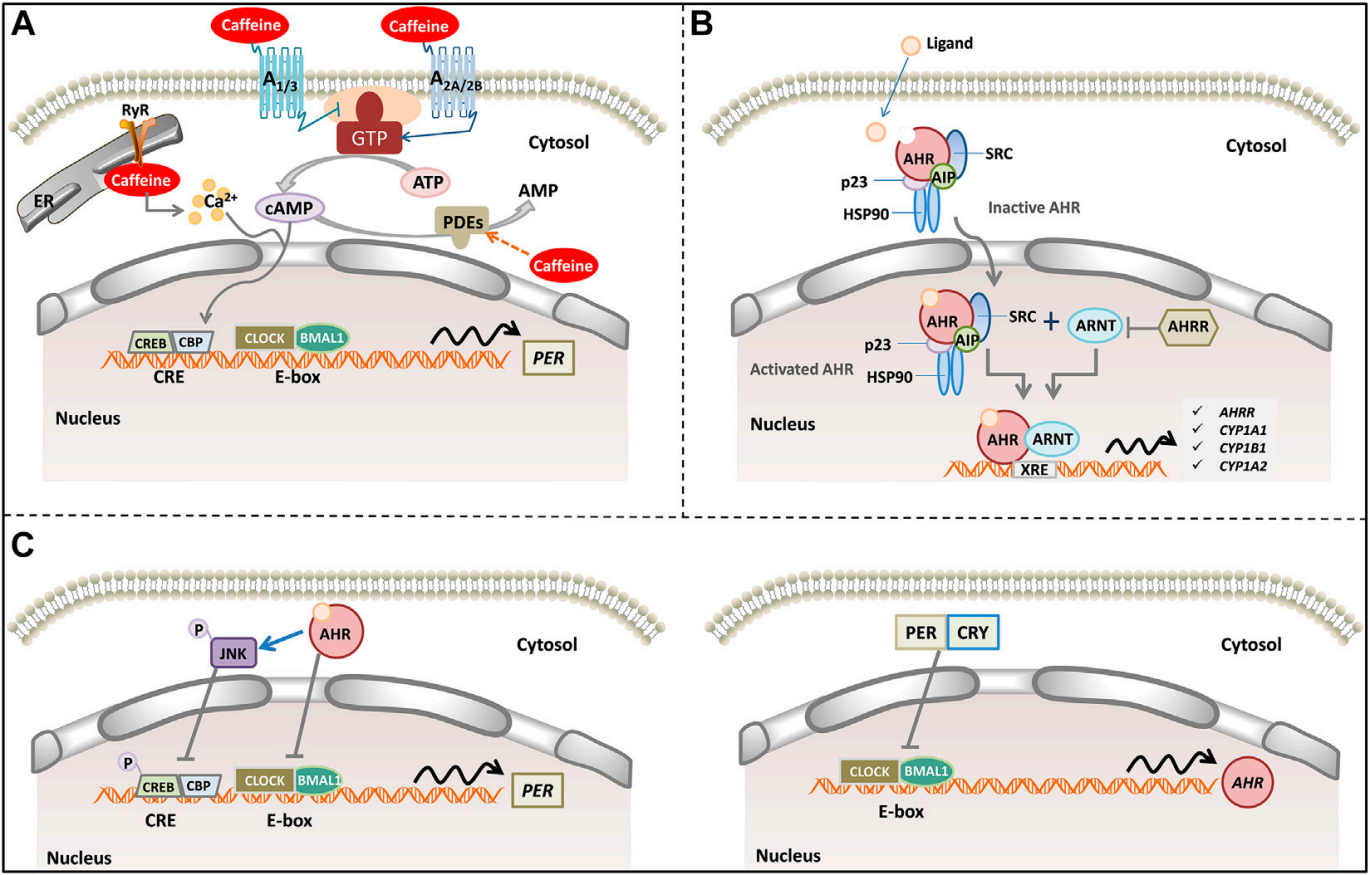
A model of caffeine, AR signaling pathway, AHR signaling pathways, and human circadian clock. **(A)** Caffeine alters intracellular cAMP levels through binding to adenosine receptors (A_1_, A_2A_, A_2B_ and A_3_) and inhibiting phosphodiesterases. In addition, caffeine mobilizes intracellular Ca^2+^ from the endoplasmic reticulum through activation of the RyR channels. Increased cytosolic cAMP/Ca^2+^ signaling culminates CREB activation acting in tandem with rhythmic transcriptional activation by CLOCK/BMAL1. CLOCK/BMAL1 binds to E-box elements in the PER promoter, where they act to stimulate *PER* transcription. **(B)** Inactive AHR complexes with HSP90, AIP, p23 and SRC in the cytosol. Upon agonist binding, AHR and some components of the chaperone complex translocate to the nucleus, where AHR forms a dimer with ARNT binding to the XRE to control gene expression (such as AHRR, CYP1A1, CYP1A2 and CYP1B1). **(C)** Activated AHR can physically interact with BMAL1, which exhibits high homology with ARNT, thereby reducing CLOCK-BMAL1 interactions and repressing PER transcription. In addition, activated AHR phosphorylates and activates JNK, which represses CRE-mediated transcriptional activity to suppress PER transcription (left). Rhythmic transcription of AHR is driven by CLOCK: BMAL1 at E-Box promoter elements. PER and CRY inhibit AHR transcript levels (right). Abbreviation: cAMP, cyclic adenosine monophosphate; RyR, ryanodine receptor; PDEs, phosphodiesterases; AHR, aryl hydrocarbon receptor; AHRR, AHR repressor; ARNT, AHR nuclear translocator; XRE, xenobiotic response element; HSP90, 90 kDa heat shock protein; AIP, AHR-interacting protein. CLOCK, circadian locomotor output cycles kaput; BMAL1, brain and muscle ARNT-Like 1; CREB, cAMP response element-binding; CBP, CREB binding protein; PER, periods; CRY, crypto-chromes.

One of the major strengths of the present study was our ability to assess how genetics affect the response to caffeine based on 147 candidate SNPs in more than 40 human genes (Table 1), encoding various proteins related to the disposition of caffeine and/or pharmacological mechanisms of caffeine’s actions, as well as circadian control functions. Along with our previous report (He et al., 2021), we observed significant associations between *ADORA1*, *ADORA2A*, *ADORA3*, *PDE4D*, *ADA*, *AHR*, and *CLOCK* polymorphisms and the response to caffeine treatment (Tables 2, 3). Notably, the haplotypes of *AHR, CLOCK*, and *ADORA2A* were found to be associated with a better response to caffeine therapy (Table 4). A_2A_-AR regulates phrenic nerve activity, and blockage with caffeine may be one mechanism responsible for the efficacy of xanthine against AOPs. Additional A_1_-AR in the brainstem may regulate hypoxic ventilator drive. Such central stimulation results in increased respiratory drive, increased sensitivity to hypercarbia, decreased hypoxic suppression of respiration, and increased diaphragmatic contractility (Morton and Smith, 2016). Caffeine competitively inhibits PDEs, a group of enzymes that degrade cAMP (Burke et al., 2015). In addition, intracellular adenosines undergo metabolism to inosine by ADA, a deamination that occurs preferentially under pathological conditions featuring raised adenosine levels (Borea et al., 2018). Collectively, caffeine may play a critical role in AOP treatment by directly and/or indirectly agonizing the adenosine-AR-cAMP pathway, rather than the AHR-CYP1A2 pathway involved in the disposition of caffeine, an XRE-dependent control of the target gene expression pathway by AHR. One outstanding question is how to explain *ADORA*, *AHR*, and *CLOCK* gene polymorphisms that were observed to be associated simultaneously with the response to caffeine therapy. However, whether the AR, AHR, and CLOCK signaling pathways crosstalk with each other during caffeine treatment remains unclear. The potential model relationship between them is discussed below and illustrated in Figure 3.

Adenosine is a ubiquitous endogenous autacoid and functions as a signaling molecule through the activation of four distinct ARs (*i.e.*, A_1_, A_2A_, A_2B_, and A_3_) (Borea et al., 2018). Adenosine encodes a sleep history and modulates circadian entrainment by light. However, caffeine has the opposite effect on sleep as adenosine, and it promotes wakefulness via A_2A_-AR, rather than A_1_-AR (Huang et al., 2005). Additionally, caffeine inhibits PDEs or promotes intracellular Ca^2+^ release (Kumar and Lipshultz, 2019). Pharmacological and genetic approaches demonstrate that adenosine acts on the circadian clock via A_1_-AR/A_2A_-AR signaling through the activation of the Ca^2+^–ERK–AP-1 and CREB/CRCT1–CRE pathways to regulate the clock genes *PER 1* and *PER 2* (Figure 3A). Interestingly, adenosine integrates light and sleep signaling for regulating the circadian rhythms in mice (Jagannath et al., 2021).

AHR is a ligand-activated transcription factor that is a member of the periodic circadian protein (PER)–AHR nuclear translocator (ARNT)–single-minded protein (SIM) superfamily of transcription factors, in which the PER–ARNT–SIM (PAS) domain senses both the endogenous and exogenous factors (Figure 3B). AHR senses oxygen levels, redox potential, and changes in the circadian rhythm and control adaptation in the cellular environment (Rothhammer and Quintana, 2019). AHR is involved in controlling the cardiovascular and respiratory functions (Sauzeau et al., 2011). There is high constitutive AHR expression in lung epithelial/endothelial cells and fibroblasts (Rico de Souza et al., 2021). AHR promotes lipid droplet biogenesis and metabolic shift in respiratory club cells, which are metabolically active cells that are involved in xenobiotic metabolism and host defense as well as the maintenance of airway integrity (Wang et al., 2021). AHR deficiency enhanced airway inflammation and remodeling in a murine chronic asthma model (Chang et al., 2020), and also caused the development of chronic obstructive pulmonary disease (Guerrina et al., 2021), indicating its importance as a central player in maintaining normal lung function and determining disease severity. In addition, AHR activity in both endothelial and hematopoietic cells is necessary for vascular development and closure of the ductus venosus (Guerrina et al., 2018). Therefore, the AHR signaling pathway may play a critical role in AOP and BPD development, as well as in the response to caffeine treatment in preterm infants.

Intrinsic clocks determine nearly all circadian cycles, such as respiratory and exercise capacity (Allada and Bass, 2021). More importantly, AHR and the circadian signaling pathways are highly integrated and reciprocally regulated (Figure 3C). AHR exhibits a rhythmic expression and time-dependent sensitivity to activation by AHR agonists. Conversely, AHR influences the amplitude and phase of rhythms in circadian clock genes, hormones, and behavior (Tischkau, 2020). AHR directly interacts with the core circadian clock. In the canonical signaling pathway, activated AHR forms a heterodimer with ARNT, which exhibits sequence homology with BMAL1. However, activated AHR can also heterodimerize with BMAL1, thereby disrupting the normal binding of CLOCK/BMAL1 to E-box elements to drive the transcription of the target genes (Jaeger et al., 2017). As one of the potential mechanisms, AHR-CLOCK might set a pathological precondition for the efficacy of caffeine treatment to manifest. Thus, investigation into the mechanism by which the AHR-CLOCK interaction affects the response to caffeine treatment may provide insights into the pathological mechanisms of AOP and provide an innovative strategy to reset the treatment protocol in preterm neonates.

However, our study has several limitations. In this study, only the *CLOCK* and *BMAL1* polymorphisms were assessed for their potential roles in the circadian rhythm. In mammals, at the cellular level, the transcription factors CLOCK and neuronal NPAS2 form heterodimers with ARNTL1 (*i.e.*, BMAL1) to drive the expression of the genes encoding period circadian protein homologues PER1, PER2, and PER3 and crypto-chromes (CRY) 1 and 2 via direct binding to the E-box enhancer element during the day. In the late afternoon or evening, PER and CRY proteins heterodimerize, translocate into the nucleus, and interact with CLOCK and BMAL1, thus suppressing their transcriptional activity. In the meantime, the protein levels of PER1, PER2, CRY1, and CRY2 also decline by poly-ubiquitination and subsequent degradation via specific E3 ligase complexes (Ruan et al., 2021). However, we did not determine the roles of the genes encoding PER and CRY proteins in caffeine therapy. Another limitation is that this study was performed in a single center with a small sample size (n = 112), which may increase the risk that some significant changes will not be captured. The missed significant association between CYP1A2*1B (rs2470890) variant and response to caffeine therapy (*p* = 0.058; codominant model) in preterm babies may be due to the small study cohort. In addition, as described in our previous report (He et al., 2021), the grouping criteria may limit the reliability and accuracy of the present study. Lastly, in terms of statistical approach, within an FDR threshold of <0.05, 5% of the variants (*i.e.,* 4-5 SNPs) were estimated to be false positives, which was generally acceptable.

## Conclusion

Our study found that candidate *CYPs 1A2*, *3A4*, *3A5*, *3A7*, *3A43, AHRR*, *ARNT*, and *BMAL1* polymorphisms had no effect on the response to caffeine therapy in the apnea-free and apneic groups. However, genetic candidate variants in *AHR* and *CLOCK* genes were found to be associated with variable responses to caffeine treatment in these preterm babies. Moreover, the variants in *AHR*, *ADORA2A*, and *CLOCK* genes formed strong LD with increased ORs, which were associated with a significantly better response to standard-dose caffeine therapy. Future and larger studies, as well as basic research, are required to understand how these SNPs affect the response to caffeine therapy. Our findings also indicate that circadian rhythm may play an essential role in the response to caffeine therapy in babies experiencing AOP episodes.

## What is Already Known About This Subject

• Caffeine citrate therapy is a preferable choice for preterm infants with AOP in the NICU.
• The current standard-dose caffeine therapy leads to variable clinical outcomes. It remains unclear why some preterm infants respond well to this therapy but others do not.
• Questions regarding dose selection, routine TDM of caffeine, and influence of genetic variants are still unanswered.

## What This Study Adds

• No significant intergroup differences in allele distributions or genotype frequencies of *CYP1A2*, *CYP3A4*, *CYP3A5*, and *CYP3A7* were found in our study on preterm babies.
• To the best of our knowledge, the present study is the first to report that *CLOCK* gene polymorphisms are involved in determining the response to caffeine therapy in premature neonates with AOP.
• Strong linkage disequilibrium was observed in *AHR*, *ADORA2A*, and *CLOCK* variants. These genetic variants were significantly associated with a better response to standard-dose caffeine therapy. However, whether the AR, AHR, and CLOCK signaling pathways crosstalk with each other during caffeine treatment remains largely unknown.

## Data Availability

The original contributions presented in the study are included in the article/Supplementary Material, further inquiries can be directed to the corresponding authors.
