# Supplementary material for: Caffeine Therapy for Apnea of Prematurity: Role of the Circadian CLOCK Gene Polymorphism
Source: Front Pharmacol. 2022 Jan 25;12:724145. doi: 10.3389/fphar.2021.724145 (PMC8822171; doi:10.3389/fphar.2021.724145)
Supplement: Supplementary file 1 [file DataSheet1.pdf]

Caffeine Therapy for Apnea of Prematurity: Role of the Circadian *CLOCK* Gene Polymorphism

Hong-Li Guo[1, †], Jia-Yi Long[1,2,†‡], Ya-Hui Hu[1], Yun Liu[3], Xin He[1], Ling Li[1,2,‡], Ying Xia[1], Xuan-Sheng Ding[2], Feng Chen[1,*], Jing Xu[1,*], Rui Cheng[3,*]

[1]Pharmaceutical Sciences Research Center, Department of Pharmacy, Children's Hospital of Nanjing Medical University, China

[2] School of Basic Medical Sciences and Clinical Pharmacy, China Pharmaceutical University, China

[3] Neonatal Intensive Care Unit, Children's Hospital of Nanjing Medical University, Nanjing, China

[†]These authors contributed equally to this work.

[‡]Visiting graduate student from China Pharmaceutical University.

*Corresponding authors at Children's Hospital of Nanjing Medical University, 72 Guangzhou Road, Nanjing 210008, China.

E-mail addresses: cy.chen508@gmail.com (F. Chen), njxujing@163.com (J. Xu), chengrui350@163.com (R. Cheng).

----Supplemental information----

Table 1 Genotyping information for 88 selected single nucleotide polymorphisms (SNPs)

Gene	SNP-ID	1st-PCRP	2nd-PCRP	Amplification length (bp)	UEP_SEQ
AHR	rs10249788	ACGTTGGATGTGCCCCCGCCCATCTGGATT	ACGTTGGATGCTTACGTCCTACGTCATCAC	101	GGCCCTCAAGGAAGA
AHR	rs10250822	ACGTTGGATGCTCCTTTATCTACTTTCCTG	ACGTTGGATGGACATAAATCCTGTCTACTG	98	GTCTACTGATACATTATAAAGTCT
AHR	rs1476080	ACGTTGGATGACCACTATTCGGTATTCCTC	ACGTTGGATGCTCCCTAAATGCCACATCTC	104	ggacTGCCACATCTCTTCATATC
AHR	rs2066853	ACGTTGGATGCCTAGGCATTGATTTTGAAG	ACGTTGGATGTCTGAAGTCAACCTCACCAG	105	gggcAAAATTTTTCATTCTGCATGTGT
AHR	rs2158041	ACGTTGGATGCTTGATGCCAACCATTACAC	ACGTTGGATGTGAAGGGTTTTGTTACAGGG	106	GTGTTTCAGATGCAACTTT
AHR	rs6960165	ACGTTGGATGGAATGAACAGTGAATAGATGG	ACGTTGGATGGTTACAATCTGTTCTCAAACC	107	CAAACCATAAAAGATGTTCTTG
AHR	rs7811989	ACGTTGGATGCAAGTCCACTCTGAGGAATC	ACGTTGGATGTCTGAACACCTGCATAGGAG	82	gGGAGTTGACTGTAGGGA
AHRR	rs2292596	ACGTTGGATGGGTTTTGCTGACTCACTTGG	ACGTTGGATGACCCTCCCCAGGTGGTGTTT	100	AGGTGGTGTTTGGGCAG
ARNT	rs2228099	ACGTTGGATGATTCAGACTGTGGCTGGTTC	ACGTTGGATGTTGTCTCATGTGAGACAGGC	98	GCAGGGTGGTGTATGT
BMAL1	rs11022775	ACGTTGGATGCTAAGAAGCAGAGTCCTCAG	ACGTTGGATGGGACTTCATACTTTGCCCTC	101	TCTCCTCAAACTTCCATC
BMAL1	rs1122780	ACGTTGGATGCTAGCAACATACCTGTTGTG	ACGTTGGATGCTTTATCCTTGAGTCATTC	115	CTTGAGTCATTCATATTTTTAGTG
BMAL1	rs1481892	ACGTTGGATGCCCTTCACACCAACAATCTC	ACGTTGGATGGCAGTTTTGTTAATACAGCC	107	CAAATCTTTCTATGGTGCTT
BMAL1	rs1868049	ACGTTGGATGGCACTATATTGGGTTGTGGG	ACGTTGGATGGTTTGCCACACACACTTTCC	100	ttcatAGCCACATGCATTTGCT
BMAL1	rs2278749	ACGTTGGATGAACCCCACCTATGGTAAGAG	ACGTTGGATGATCATTACAGCACCTCCTCC	100	ggaagGCAGTGCACCTAGCCCC
BMAL1	rs2279287	ACGTTGGATGAGTAGCCAGGTTTTGACCAC	ACGTTGGATGCCGTAGACAGCTTTTTGCAG	101	cccccGCCGCGGGGCTGGTGTCTAC
BMAL1	rs2290035	ACGTTGGATGGGAGTTACAAAAAGAAAGGC	ACGTTGGATGTTTTTCTCCCCTGGGCTTTC	97	gttgTTTTCCTAAACTCTCCCT
BMAL1	rs3816358	ACGTTGGATGTTTCTGGCTGGCCAGAGTG	ACGTTGGATGTTTTCAGCTCTCTCCTCTGC	119	CCTCTGCTCCCAACT
BMAL1	rs3816360	ACGTTGGATGCAACCAGTGAATGAGGGCAG	ACGTTGGATGATTGACAGTCATGGGCACAG	109	cctcAAAGGTCCTTTAGAAAGTTGTGGC
BMAL1	rs4757142	ACGTTGGATGTCAGTCCACTCTCAACACAG	ACGTTGGATGTTTGAAGGAGGAACGACAGG	95	tgagtAGGAACGACAGGATCTGAGGCAC
BMAL1	rs4757144	ACGTTGGATGGGTTTCTCTAGACTGTAGGC	ACGTTGGATGTAGTGTTCTAGGCAGTTGGG	103	aagaACGGGGTGAATTTAACAG

BMAL1	rs6486120	ACGTTGGATGAGAGGCCTCTTGCATCATGG	ACGTTGGATGTGGAAAATTGGGCCCATGTG	114	cGGCTCTGTGTGTGGTTTAT
BMAL1	rs7126303	ACGTTGGATGAAAAGGGTCACCCCACATCC	ACGTTGGATGGGAAAGCCCCGTTTAAAATC	101	GCCATCTCAAGGCAGTG
BMAL1	rs969485	ACGTTGGATGATGTGAGTGGAAGAAGGGAG	ACGTTGGATGGGAACTCTTGAATTGCTCCC	100	gctccATTAAATGAACTGACAGTTCC
CLOCK	rs10002541	ACGTTGGATGTTCGGAGCTTTCCAATTGCC	ACGTTGGATGAGAAACTGGGTCTTCTGGTG	94	tgccgGGGTCTTCTGGTGTCAATG
CLOCK	rs10462028	ACGTTGGATGATGACAAAGGAGAGCACAGC	ACGTTGGATGATACTACGGTCTAGCCTTCG	92	TCGAGCATCCCAAATTC
CLOCK	rs1048004	ACGTTGGATGGATAGTGTTAGGTTATCATC	ACGTTGGATGGACTGCTATCAGTCTCTTGG	90	gGTCTCTTGGACTGGAG
CLOCK	rs11133373	ACGTTGGATGCCCATTTCTGCAAATAAGGC	ACGTTGGATGTTTCAGGTTTTACTCTTGCC	107	cTACTCTTGCCTCCGTAG
CLOCK	rs11133385	ACGTTGGATGTCAAGCTCCCTTCGTCTATG	ACGTTGGATGGTGATGGGAAGAGAAGAGAC	98	ggGAGAAGAGACCAAATTGTTAA
CLOCK	rs11133389	ACGTTGGATGGGATCAGTAATCAGAATGGC	ACGTTGGATGCTGAATGCTGAAGACTCCAC	98	tatCTTCCAGTGTGGTGT
CLOCK	rs11133391	ACGTTGGATGGGGAAATTACAAATATACAC	ACGTTGGATGGATTGATTAGCCATGAGTTG	119	cagtGAAACTGGGTGAAGGATAT
CLOCK	rs11240	ACGTTGGATGAGATGAACAGATGAGACACG	ACGTTGGATGTTTTAGTGTCTGGTCATTCG	99	TCTGGTCATTCGTTTAAAATA
CLOCK	rs11726609	ACGTTGGATGTGGTGGTCTTCATCTTGTTC	ACGTTGGATGCCCAGCAGACAGAATAGTAG	99	gatAATAGTAGCTGGCACAGAACAGAA
CLOCK	rs11735267	ACGTTGGATGATTGGTGGCTAACTCAACCC	ACGTTGGATGGGCTAAAGGAGTGAATAGGG	109	TAAAGGAGTGAATAGGGAATTTAT
CLOCK	rs11824092	ACGTTGGATGCAGTCATTTGTCCATCCACC	ACGTTGGATGAGCACTCTAGGGCCTAAAAC	100	taCTGGCACACAGTGGG
CLOCK	rs11931061	ACGTTGGATGGTTACTCTAACTCCCAGCTC	ACGTTGGATGAATCCTTGTATCTGCAGCAC	102	ccctgATCTGCAGCACTAGTGTAGC
CLOCK	rs11932595	ACGTTGGATGATGAGTTGAAGGACAGGGAG	ACGTTGGATGCATCCATCTTGAGTGCATTG	104	GTTTAGACCCCTGCC
CLOCK	rs11943456	ACGTTGGATGCCCTCCTTTTCTCTTTCTTC	ACGTTGGATGGCAAGTTACTGAACCAAAACC	117	aatgCTGAACCAAAACCCAGCCT
CLOCK	rs12504300	ACGTTGGATGACCCTTTTCCCAAGAGTATG	ACGTTGGATGGCATCTTAGCATTTGTGACTG	114	ATTTGTGACTGATAAGAAGTAA
CLOCK	rs12648271	ACGTTGGATGCCATCTGTAAACAGTCCAGT	ACGTTGGATGGTCTTTTGTTGACTTCGTAAG	119	cTATTGTAGTTATGTTTTATAAAAGCC
CLOCK	rs12649507	ACGTTGGATGCATATCCCAATACCAACACC	ACGTTGGATGGGGACATAACAGCCTAAATG	83	GCCTAAATGCAGTAAAGAG
CLOCK	rs13102385	ACGTTGGATGCATGACACAGCATGTACTTC	ACGTTGGATGGAGCTGAAAAATTATTGCCC	114	gttgACTGTATATTGGTAACCTGG
CLOCK	rs13132420	ACGTTGGATGTCATACTACTGTGTCTGCCC	ACGTTGGATGCCCGGCCTAGAGCTTTTTTT	99	GCCTAGAGCTTTTTTTTTCCTTA
CLOCK	rs17721497	ACGTTGGATGACACTGCCAAGAAAGTGCTC	ACGTTGGATGTAAAAGCTTCCCGTTGGCAC	97	GTTGGCACCCTGACA
CLOCK	rs1801260	ACGTTGGATGTAAATACCAGCCAGCAGGAG	ACGTTGGATGCAGGCACCTAAAACACTGTC	86	ggaaCTAAAACACTGTCAGAACTGGCT

CLOCK	rs2070062	ACGTTGGATGCCTTTTGTACTCCTTGGATCT	ACGTTGGATGATTCTGAGACTTATGGTTGG	112	TGGTTGGTCATATAGAAGAG
CLOCK	rs2272073	ACGTTGGATGTTACGGTTGAGTTCCCAGAG	ACGTTGGATGTAGAACATTTATTGCGAGCC	118	TTGCGAGCCTCCTCTA
CLOCK	rs2412646	ACGTTGGATGGGAGTAAGTTGTTACACAGC	ACGTTGGATGAGTCAGAATTGGAACTCTGG	110	TGTTATATCAGGTCAGAAGT
CLOCK	rs2412648	ACGTTGGATGCTAGCTGGGTAACTTTGGAC	ACGTTGGATGACCGCCTCTCACTCTCTAAG	91	TCTCACTCTCTAAGGAGGAAA
CLOCK	rs3736544	ACGTTGGATGGGTTCCGTTCAACTTTCTTC	ACGTTGGATGTAGGAACAACTTGGCCTTGC	102	tatcGGTGCAAGTTGCTGGAT
CLOCK	rs3749474	ACGTTGGATGAATCAAAACTCCCAGCCACC	ACGTTGGATGGTTATTCAGAAATATCCCTC	114	ggCCCTCATTTCATTATTTTTCAATTAT
CLOCK	rs3762836	ACGTTGGATGACGGACACGCATGATAGAAG	ACGTTGGATGCCATGGACCATCTGAAGTTG	104	ccTTCTTCTTGTTGCCGA
CLOCK	rs3792603	ACGTTGGATGCTGGCCTCAGAGTATTTGTT	ACGTTGGATGCACACACGTTCTTTCACTTT	120	GTTCTTTCACTTTATGTCCT
CLOCK	rs3805147	ACGTTGGATGGCATTAAAGTATTTGGTTG	ACGTTGGATGCACTTAGTACAGATCTTGAC	87	TCTTGACAATAAAATACTCTATTTTATA
CLOCK	rs3805148	ACGTTGGATGGTCCATATTAAGAAAGTCC	ACGTTGGATGGCACTTAGTACTTCTGTGAC	92	gGTGACTAAGTGTACTTTGTAAATA
CLOCK	rs3805151	ACGTTGGATGGACAGAACTTTGGATATTTC	ACGTTGGATGAAAGTATTTTGCAGGACAG	98	ggTGCAGGACAGAAATAAAAAT
CLOCK	rs3817444	ACGTTGGATGGAATTTGCAGTGTACCGGTG	ACGTTGGATGAAAGCTGCCAAGGAAAGTAG	105	cctgaCAAGGAAAGTAGCAGATCATT
CLOCK	rs4340844	ACGTTGGATGCATTAGGCCACTCTAGTCTG	ACGTTGGATGTCCATTCCATCCATGGCTTC	111	cccgAGGTATCTATTATATACAGCCTA
CLOCK	rs4580704	ACGTTGGATGGCACTAAATTGGCAGGCTTC	ACGTTGGATGTCCCATCACAAGTCCCAAAC	98	gggtTCCCAAACCTAATCTGTCAT
CLOCK	rs4864546	ACGTTGGATGAACCCAAAAGTAAGCTGAGG	ACGTTGGATGGTTTAATTGACTGCCTGTGG	94	ggagGCCTGTGGTTTTCTTATTTC
CLOCK	rs4864548	ACGTTGGATGAGGGTCCTGGTTTATTTCTG	ACGTTGGATGTGGAGATTTTTCCCTTGCCC	110	GACAGTGTAATGGAAGATG
CLOCK	rs534654	ACGTTGGATGCTCAGTGCCAGGAATCAAGG	ACGTTGGATGTTACTACATACCCCTCTCCC	111	cctcGTCTGCTTTGCTCAGGTA
CLOCK	rs6811520	ACGTTGGATGACTTACCATTTGCCTCATAC	ACGTTGGATGATAGGGAACAGGCCTTTCCA	99	GGCCTTTCCAATATAGATG
CLOCK	rs6832769	ACGTTGGATGCTACAGCACTTCTGAAGACC	ACGTTGGATGCAAGATGCCAGTGATTTGGA	104	GTGATTTGGAATAGTTTGTTATAGAA
CLOCK	rs6843722	ACGTTGGATGAGCCAACCATGGATCAAAAC	ACGTTGGATGAGTGTTTAGGGAACGATTAG	116	cagtGAACGATTAGAAAAATGCACGTA
CLOCK	rs6850524	ACGTTGGATGCCACGCTATAAATTTCCCTC	ACGTTGGATGCAACTTAACAAAATATGCGGG	82	CGGGATGCAACAAATG
CLOCK	rs6858749	ACGTTGGATGAGTCAAACAAATCCTGATG	ACGTTGGATGGCTACGTATTGCCTGTGAAG	99	acGGAAGTTTAATAACTGCTGAAAGG
CLOCK	rs726967	ACGTTGGATGCCTTCTGCAGTGTACTAATG	ACGTTGGATGGCAGAGGTGAGAAAAATATA	119	AGTAATCACAGAAGAATTCATCA
CLOCK	rs7660668	ACGTTGGATGCCTAAGTGCAGACAATTTCC	ACGTTGGATGAGGAGTTCAAGACAAGCCTG	116	TGGGCAACAAAGTCA

CLOCK	rs7698022	ACGTTGGATGGTGTAACTTCTGTGAAACAGG	ACGTTGGATGGAGGCTATAAGAGTAAGTT	120	ccacAAAATGGAAAAAAACAGGAATCTC
CLOCK	rs9312661	ACGTTGGATGAGGAGTACAAAAGCCCTACA	ACGTTGGATGGGACCTCTTGATATCTACCC	120	cctcCCCAATAAATGTTAATGCCTA
*CYP1A2*1B*	rs2470890	ACGTTGGATGGCCTCAGAATGGTGGTGTCT	ACGTTGGATGTCTACGGGCTGACCATGAAG	114	TGCGCTTCTCCATCAA
*CYP1A2*1C*	rs2069514	ACGTTGGATGAGTGCAGTGGTGCGATCTTG	ACGTTGGATGTGTAATTCCAGCTACTCGGG	112	cgggtCATGACAATTGCTTGAATC
*CYP1A2*1D*	rs35694136	ACGTTGGATGGATTGTTTGAGCTCAGGAGG	ACGTTGGATGACAGAGTCTTGCTCTGTCAC	107	ggtaACCCAGGTTGGGGTTC
*CYP1A2*1E*	rs2069526	ACGTTGGATGTAGAACCTGGAAGCTAGTGG	ACGTTGGATGTCAAGAGCTGGGTAGCAAAG	111	cccAGCCCGGAACTCAGG
CYP3A4	rs4646437	ACGTTGGATGAGGGCAGGTCTATGCATAAG	ACGTTGGATGCTTCAAAAGATGCACAAGGG	90	TGATCTCACTGCTGTAG
*CYP3A4*18A*	rs28371759	ACGTTGGATGCACTGCTCGTGGTTTCATAG	ACGTTGGATGCTTTCCTCTCCTTTCAGCTC	109	gttgCAGCTCTGTCCGATC
*CYP3A4*1B,*	rs2740574	ACGTTGGATGATGAGGACAGCCATAGAGAC	ACGTTGGATGATCAGAAACTCAAGTGGAGC	101	TCTATTAAATCGCCTCTCTC
*CYP3A4*23*	rs2687116	ACGTTGGATGAGTGGATGAATTACATGGTG	ACGTTGGATGGTATGTGGACTACTATTTC	110	TGGACTACTATTTCCTTTTATTTATCTT
*CYP3A4*23*	rs3735451	ACGTTGGATGTACTGCATTTTTTTTGCCC	ACGTTGGATGGAGACACTCCTTCAGTGTTG	101	gggCAACAGAGTGATATTCTGATCTC
*CYP3A4*4*	rs55951658	ACGTTGGATGGTTGGAGACAGCAATGATCG	ACGTTGGATGACTCTAGCCTTTTGGTCCAG	116	aAGTGGGATTTATGAAAAGTGCC
*CYP3A43*2A*	rs61469810	ACGTTGGATGCCAGGAATTCCCAGCTTCTT	ACGTTGGATGTTCTGTAACCTGGCTTTCTC	101	CTGGCTTTCTCTTTTATTTTATAGTT
*CYP3A43*3*	rs680055	ACGTTGGATGAACTGCAGGAGGAGATTGAC	ACGTTGGATGATTCTTGCTGAGGCTTCACC	112	tctaATCCCCTTACCTTATTGG
*CYP3A5*4*	rs56411402	ACGTTGGATGGCATGGATGTGATTACTGGC	ACGTTGGATGGGAACTTCTTAGTGCTCTCC	113	TCCACAAAGGGGTCT
*CYP3A5*5*	rs55965422	ACGTTGGATGACCTGTCCCCAGATTCATTC	ACGTTGGATGAACCTTCACCAGCGGAAAAC	105	gactgCACCAGCGGAAAACTCAAGGAGG
CYP3A7	rs1021	ACGTTGGATGAAGGCGGTACTAGTTCAGAC	ACGTTGGATGGGTGAAATGTAGAATAAGGC	119	ccccATAAGGCCTTCAACTTTTTTT
CYP3A7	rs12360	ACGTTGGATGTTGAGGTCTCTGGTGTTCTG	ACGTTGGATGCGTAAGTGGAGCCTGATTTC	100	ggggaACTTCTGGTTTGCTCTT
*CYP3A7*1D*	rs55798860	ACGTTGGATGACAGCCTCACTGAATCACTG	ACGTTGGATGGTCTTTTTTTCAGCAGCGTG	107	tgcgGTGTGTGTGGAGCTTTCCTGC
*CYP3A7*2*	rs2257401	ACGTTGGATGCTTTCAGGGAGGAACTTCTC	ACGTTGGATGGTGGTGGTGATGATTCCAAG	100	cTCATGACCCAAAGTACTGGA

Abbreviations: *AHR,* aryl hydrocarbon receptor; *AHRR*, aryl-hydrocarbon receptor repressor; *ARNT*, aryl hydrocarbon receptor nuclear translocator; *BMAL1*, aryl hydrocarbon receptor nuclear translocator-like protein 1 or Brain and Muscle ARNT-Like 1; *CLOCK*, circadian Locomotor Output Cycles Kaput.

Table 2 Single nucleotide polymorphisms (SNPs) association with preterm infants in bronchopulmonary dysplasia (BPD) group and BPD-free group[†]

Gene	SNPs	Model	Genotype	Frequency no. (%)	BPD group no. (%)	BPD -free group no. (%)	0dds ratio	95% CI	*p*
ADORA1	*rs16851030 C>T*	Dominant	CC	45 (40.91)	17 (58.6)	28 (34.6)	1		0.025
			TC/TT	65 (59.09)	12 (41.4)	53 (65.4)	2.68	1.12-6.40	
ADORA2A	*rs2236625 G>A*		CC	92 (82.88)	28 (96.5)	64 (78)	1		0.01
			TC/TT	19 (17.12)	1 (3.5)	18 (21.9)	7.87	1.00-61.91	
	rs34923252 T>A		TT	94 (83.93)	28 (96.5)	66 (79.5)	1		0.015
			TA/AA	18 (16.07)	1 (3.5)	17 (20.5)	7.21	0.91-56.85	
	rs5760425 T>G		TT	31 (27.93)	4 (13.8)	27 (32.9)	1		0.038
			GT/GG	80 (72.07)	25 (86.2)	55 (67.1)	0.33	0.10-1.03	
	rs5996696 A>C		AA	94 (83.93)	28 (96.5)	66 (79.5)	1		0.015
			CA/CC	18 (16.07)	1 (3.5)	17 (20.5)	7.21	0.91-56.85	
	rs2298383 C>T		CC	31 (27.93)	4 (13.8)	27 (32.9)	1		0.038
			CT/TT	80 (72.07)	25 (86.2)	55 (67.1)	0.33	0.10-1.03	
	rs4822492 C>G		CC	79 (71.82)	4 (13.8)	27 (33.3)	1		0.035
			CG/GG	31 (28.18)	25 (86.2)	54 (66.7)	0.32	0.10-1.01	
	rs5751876 T>C		TT	31 (27.93)	4 (13.8)	27 (32.9)	1		0.038
			CT/CC	80 (72.07)	25 (86.2)	55 (67.1)	0.33	0.10-1.03	
	rs5760423 T>G		TT	31 (27.93)	4 (13.8)	27 (32.9)	1		0.038
			GT/GG	80 (72.07)	25 (86.2)	55 (67.1)	0.33	0.10-1.03	
ADORA3	*rs10776727 C>A*		CC	40 (36.36)	6 (20.7)	34 (42)	1		0.035
			CA/AA	70 (63.64)	23 (79.3)	47 (58)	0.36	0.13-0.98	
BMAL1	*rs4757144 G>A*		GG	38 (34.55)	5 (18.5)	33 (39.8)	1		0.036
			AG/AA	72 (65.45)	22 (81.5)	50 (60.2)	0.34	0.12-1.00	
PDE1A	*rs1549870 G>A*		GG	54 (49.54)	19 (65.5)	35 (43.8)	1		0.043
			AG/AA	55 (50.46)	10 (34.5)	45 (56.2)	2.44	1.01-5.91	
PDE4A	*rs6511698 C>T*		CC	27 (24.32)	3 (10.3)	24 (29.3)	1		0.03
			TC/TT	84 (75.68)	26 (89.7)	58 (70.7)	0.28	0.08-1.01	
ADORA2A	*rs4822489 G>A*	Codominant	GG	34 (30.36)	7 (24.1)	27 (32.5)	1		0.028
			GT	47 (41.96)	18 (62.1)	29 (34.9)	0.42	0.15-1.16	
			TT	31 (27.68)	4 (13.8)	27 (32.5)	1.75	0.46-6.68	
AHR	*rs7811989 G>A*		GG	67 (59.82)	13 (44.8)	54 (65.1)	1		0.0054
			AG	38 (33.93)	16 (55.2)	22 (26.5)	0.33	0.14-0.80	
			AA	7 (6.25)	0 (0)	7 (8.4)	NA	0.00-NA	
PDE4D	*rs966221 A>G*		AA	62 (55.86)	17 (58.6)	45 (54.9)	1		0.019
			AG	44 (39.64)	8 (27.6)	36 (43.9)	1.7	0.66-4.39	
			GG	5 (4.5)	4 (13.8)	1 (1.2)	0.09	0.01-0.91	
BMAL1	*rs2290035 T>A*		TT	46 (48.94)	8 (33.3)	38 (54.3)	1		0.0006
			AT	36 (38.3)	16 (66.7)	20 (28.6)	0.26	0.10-0.72	
			AA	12 (12.77)	0 (0)	12 (17.1)	NA	0.00-NA	
CYP1A2	*rs762551 A>C*		AA	49 (44.14)	17 (58.6)	32 (39)	1		0.033
			AC	45 (40.54)	6 (20.7)	39 (47.6)	3.45	1.22-9.78	
			CC	17 (15.32)	6 (20.7)	11 (13.4)	0.97	0.31-3.09	
	rs2472299 G>A		GG	49 (44.14)	17 (58.6)	32 (39)	1		0.033
			AG	45 (40.54)	6 (20.7)	39 (47.6)	3.45	1.22-9.78	
			AA	17 (15.32)	6 (20.7)	11 (13.4)	0.97	0.31-3.09	

† Data from BPD group was defined as case group and data from BPD-free group was defined as control group for the association analysis.

Abbreviations: BPD, bronchopulmonory dysplasia; *ADORA1*, Adenosine A1 receptor gene; *ADORA2A*, Adenosine A2A receptor gene; *ADORA3*, Adenosine A3 receptor gene; *BMAL1*, aryl hydrocarbon receptor nuclear translocator-like protein 1 or Brain and Muscle ARNT-Like 1; *PDE1A*, Phosphodiesterase 1A gene; *PDE4A*, Phosphodiesterase 4A gene; *PDE4D*, Phosphodiesterase 4D gene; *AHR*, Aryl hydrocarbon receptor gene; CI, Confidence interval.

Table 3 Single nucleotide polymorphisms (SNPs) association with preterm infants in severe neurological injury (SNI) group and SNI-free group[†]

Gene	SNPs	Model	Genotype	Frequency no. (%)	SNI group no. (%)	SNI-free group no. (%)	0dds ratio	95% CI	*p*
ADA	*rs2472304 G>A*	Dominant	GG	79 (71.17)	11 (52.4)	68 (75.6)	1		0.041
			GA/AA	32 (28.83)	10 (47.6)	22 (24.4)	0.36	0.13-0.95	
ADORA2A	*rs5760410 A>G*	Codominant	AA	32 (29.36)	8 (36.4)	24 (27.6)	1		0.043
			GA	53 (48.62)	13 (59.1)	40 (46)	1.03	0.37-2.83	
			GG	24 (22.02)	1 (4.5)	23 (26.4)	7.67	0.89-66.20	
AHRR	*rs2292596 C>G*		CC	55 (49.55)	14 (66.7)	41 (45.6)	1		0.011
			CG	38 (34.23)	7 (33.3)	31 (34.4)	1.51	0.55-4.19	
			GG	18 (16.22)	0 (0)	18 (20)	NA	0.00-NA	
CLOCK	*rs2070062 A>C*		AA	91 (82.73)	18 (85.7)	73 (82)	1		0.01
			CA	17 (15.45)	1 (4.8)	16 (18)	3.95	0.49-31.74	
			CC	2 (1.82)	2 (9.5)	0 (0)	0	0.00-NA	
BMAL1	*rs4757142 G>A*		GG	49 (44.14)	10 (47.6)	39 (43.3)	1		0.032
			AG	48 (43.24)	5 (23.8)	43 (47.8)	2.21	0.69-7.02	
			AA	14 (12.61)	6 (28.6)	8 (8.9)	0.34	0.10-1.21	

[†] Data from SNI group was defined as case group and data from SNI-free group was defined as control group for the association analysis.

Abbreviations: SNI, Severe neurological injury; *ADA*, Adenosine deaminase gene; *ADORA2A*, Adenosine A2A receptor gene; *AHRR*, aryl-hydrocarbon receptor repressor; *CLOCK*, circadian Locomotor Output Cycles Kaput; *BMAL1*, aryl hydrocarbon receptor nuclear translocator-like protein 1 or Brain and Muscle ARNT-Like 1; CI, Confidence interval.
